# The neural correlates of discrete gait characteristics in ageing: A structured review

**DOI:** 10.1016/j.neubiorev.2018.12.017

**Published:** 2019-05

**Authors:** Joanna Wilson, Liesl Allcock, Ríona Mc Ardle, John-Paul Taylor, Lynn Rochester

**Affiliations:** a Institute of Neuroscience, Newcastle University Institute of Ageing, Newcastle Upon Tyne, UK; b Geriatric Medicine, Northumbria Healthcare Trust, UK; c Newcastle Upon Tyne Hospital NHS Foundation Trust, UK

**Keywords:** Neuroimaging, Gait, Older adults, Ageing

## Abstract

Gait is complex, described by diverse characteristics underpinned by widespread central nervous system networks including motor and cognitive functions. Despite this, neural substrates of discrete gait characteristics are poorly understood, limiting understanding of gait impairment in ageing and disease. This structured review aims to map gait characteristics, defined from a pre-specified model reflecting independent gait domains, to brain imaging parameters in older adults. Fifty-two studies of 38,029 yielded were reviewed. Studies showed inconsistent approaches when mapping gait assessment to neural substrates, limiting conclusions. Gait impairments typically associated with brain deterioration, specifically grey matter atrophy and white matter integrity loss. Gait velocity, a global measure of gait control, was most frequently associated with these imaging markers within frontal and basal ganglia regions, and its decline predicted from white matter volume and integrity measurements. Fewer studies assessed additional gait measures or functional imaging parameters. Future studies mapping regional neuroanatomical and functional correlates of gait are needed, including those which take a multi-process network perspective to better understand mobility in health and disease.

## Highlights

• Gait impairments are linked to deterioration of the brain.
• Evidence suggests that specific brain regions relate to different gait components.
• Future gait velocity decline may be predicted from measurements of white matter.
• Cognition can mediate associations between gait and neuroimaging parameters.
• Functional neuroimaging will aid further interpretation of neural-gait correlates.

## 1 Introduction

Gait is considered to be an important indicator of overall health; poor gait performance in older adults is associated with greater morbidity, mortality, and fall risk (Hausdorff et al., 2001; Studenski et al., 2011; Verghese et al., 2011). The idea that a fully functioning motor system is solely responsible for effective gait has been replaced by a more encompassing sensory-cognitive-motor model which reflects a contemporary understanding of gait as a highly complex skill (Montero-Odasso et al., 2012). Safe and effective negotiation of complex environments encountered in real-world settings requires the integration of external sensory information with neural networks which involve cortical, subcortical, brainstem, and spinal cord structures (Bohnen and Jahn, 2013; Takakusaki, 2013). Additionally, the control of gait from higher-order regions responsible for cognition is becoming increasingly evident, with implications for ageing, mobility and cognitive decline (Morris et al., 2016).

Understanding the mechanisms of simple gait, which does not involve obstacles or slopes, is crucial to improve the health status of the older population, yet the precise nature of neural control of gait during typical healthy ageing is currently unclear. The brain has traditionally been assessed through the definition of specific localised regions of interest; however, it is now thought more appropriate to consider regions connected together, either structurally or functionally, as defined neural networks. This integrative brain network perspective should increase our understanding of the dynamic brain during gait, and may also help to address common and overlapping neural outputs such as gait and cognition. In spite of this, whilst topological associations with different motor and cognitive domains have been established, neither precise brain regions nor neural processes or networks linked to discrete characteristics of gait control have been well defined. This lack of clarity causes difficulty in discerning whether individual neural regions or networks should be targeted in a different manner when aiming to improve gait impairments.

### 1.1 Quantitative gait analysis

**Fig. 1:**
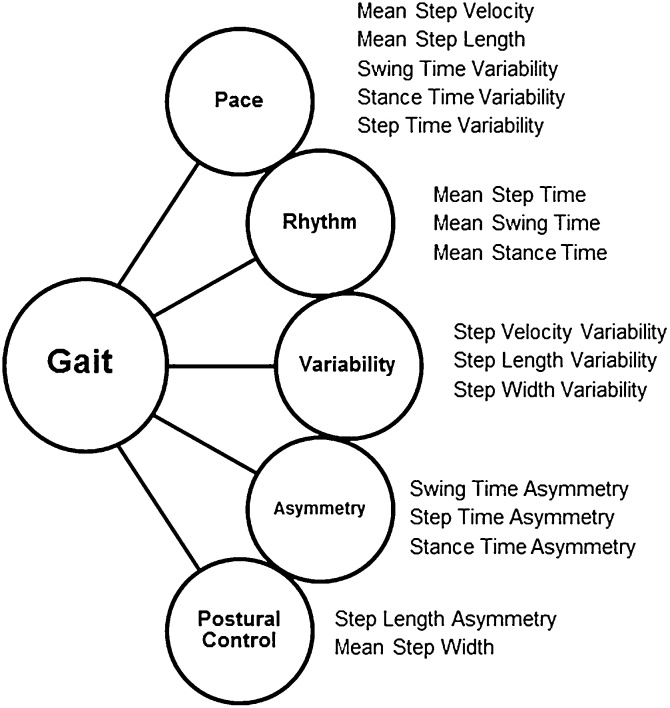
The model of gait developed by Lord et al. (Lord et al., 2013) for older adults. 16 gait characteristics map to 5 gait domains; Pace, Rhythm, Variability, Asymmetry and Postural Control.

Our knowledge of the neural underpinnings of gait has been restricted by the absence of a clear and comprehensive approach when measuring and reporting discrete gait characteristics. Quantitative gait measurement techniques enable enhanced and data rich spatiotemporal information about gait to be obtained over observation alone. Their lack of subjectivity allows for more accurate comparisons to be made between and within subjects, as well as between studies. Gait speed is typically used as a global measure of gait, due to its ease of measurement and reliability (Wade, 1992). However, this provides a limited approach to gait assessment, as it is not reflective of the subtle and selective gait alterations that occur in response to ageing and disease, which are potentially controlled by different networks (Stolze et al., 2001; Verghese et al., 2007; Lord et al., 2014). Gait can be represented by multiple discrete gait characteristics, which are thought to represent different features of neural control, and are therefore assumed to respond in a selective manner to ageing and pathology. Several groups have devised models of gait which sort discrete gait characteristics into specific gait domains through data reduction techniques (Verghese et al., 2008; Hollman et al., 2011; Lord et al., 2013; Verlinden et al., 2013). Independent domains of gait, and the characteristics contained within them, can then be hypothesised to reflect independent neuroanatomical and functional substrates. Although these models have similarities, subtle differences differentiate them. Verghese et al. produced a model containing three gait domains; pace, rhythm and variability. Two additional domains were included within the model from Lord et al., asymmetry and postural control, through the inclusion of more gait characteristics. Other models such as that from Verlinden et al. contain domains relating to more complex gait tasks such as turning. Fig. 1 shows the model from Lord et al. in older adults (Lord et al., 2013), which has been adopted in this review as the most comprehensive model for simple gait, to provide a framework for rationalising and interpreting study findings. With this in mind, a more comprehensive approach to the characterisation of gait is needed to fully understand the neural correlates of gait control, beyond that of gait speed.

### 1.2 Neuroimaging techniques used to assess gait

A second major challenge in understanding the neural underpinnings of gait is that imaging gait in real-time is not easily achieved. Several approaches have been developed to address this limitation. Electroencephalography (EEG) can be used to record electrical activity within the brain, whereas functional near infra-red spectroscopy (fNIRS) measures brain activity through haemodynamic responses in relation to neuronal behaviour; both provide real time information relating to brain activity during walking, However, these techniques can only measure superficial cortical activity, are indirect measures, and lack spatial resolution, so cannot accurately measure responses at the neuronal level. An alternative approach to understand the neural substrates of gait is to adopt cross-sectional and longitudinal study designs to explore the relationships between discrete gait characteristics and brain structure and function. These are typically assessed independently, using a range of neuroimaging techniques including both structural and functional magnetic resonance imaging (MRI, fMRI), diffusion tensor imaging (DTI), positron emission tomography (PET) and magnetic resonance spectroscopy (MRS). These techniques can be used to analyse the brain on both a global and regional level, aiding our understanding of the general imaging parameters associated with gait as well as the more specific brain areas linked to different aspects of gait control. Structural MRI can also be used for the detection of white matter hyperintensities (WMH), cerebral infarcts and cerebral microbleeds; these lesions are manifestations of subclinical cerebrovascular disease, yet are common amongst typically ageing older adults (Choi et al., 2012). Given the dynamic nature of gait, it is important to consider a more global integrative model of the brain functions which may underpin gait and other overlapping behaviours, rather than simply assigning specific specialisms to discrete brain regions. One approach is through the use of techniques such as fMRI, which can be used to assess the brain as a series of networks involved in gait control through defining areas with commonalities in activity levels across time. However, as gait and imaging assessments are dissociated in time, correlative comparisons are relied upon during analyses, limiting these detailed approaches.

### 1.3 Review aims

Overall aims of this review are to assess the global and regional neural correlates of gait in ageing, taking a detailed and comprehensive approach. By forming a matrix of associations between individual imaging parameters and gait characteristics (grouped into their appropriate gait domain as outlined by Lord et al. (Lord et al., 2013)), we aim to understand the discrete nature of gait control, underpinned by a robust model of gait, to aid our interpretation of study findings. In addition to cross-sectional studies, longitudinal study types which focus on healthy older adults will be included, so that the effects of typical ageing on neural gait correlates may be identified. Additionally, the effects of cognitive test scores, which assess higher order brain functions such as attention and memory, on neural gait correlates identified in individual studies will be outlined. This will help us to determine whether any neural pathways or regions are shared between gait and cognition, as the aforementioned cognitive control of gait suggests a three-way interplay between gait, cognitive and neuroimaging parameters. This review leads on from two recently published articles which identify links between gait and brain imaging, but with a wider range of clearly defined gait characteristics which map to a validated gait model (Tian et al., 2017a; Wennberg et al., 2017b). Our specific aims are to: i) explore associations between discrete gait characteristics and brain structure and/or function in older adults, as identified through neuroimaging; ii) explore the longitudinal relationship between changes in gait and anatomical or functional imaging correlates, and; iii) identify recommendations for future areas of research. We hypothesise that independent gait characteristics will reflect discrete regional brain structure and functional brain activity in older adults. To the best of our knowledge, no other review has taken a structured approach to comprehensively map the neural correlates of gait to a full robust gait model. This review will therefore provide a clear representation of the current literature, provide a map of the neural correlates of gait control and highlight gaps for future research.

## 2 Methods

### 2.1 Search strategy

Three databases were used for the search: Medline, PsycInfo, and Scopus. Search terms relating to gait, neuroimaging, and older adults were included within each search; where possible, age limits and MESH headings were used. The search was limited to full journal articles only, written in the English language between 1990 and April 2018. Boolean operators were utilised in the search; “OR” was included between search terms within each section, whereas “AND” was included between the sections within each database. Table 1 includes the search terms used for each search.

**Table 1:** Search terms used for the searches performed within each of the databases. All searches contained terms from the gait, neuroimaging and old age categories.

	Medline	PsycInfo	Scopus
**Gait**	**MESH headings**: Gait; Walking; Locomotion **Key words:** Speed*; Velocity*; Step*; Stride*; Rhythm; Pace; Variability; Symmetry; Asymmetry; Swing; Dual task*; Stance; Ambul*	**MESH headings**: Gait; Walking; Locomotion **Key words**: Speed; Velocity; Step*; Stride*; Rhythm; Pace; Variability; Symmetry; Asymmetry; Swing; Dual task*; Stance; Ambulation; Ambulate; Ambulatory; Ambulating	**Key word search:** Gait OR; Walk* OR; Locomotion OR; Speed OR; Velocity OR; Step* OR; Stride* OR; Rhythm OR; Pace OR; Variability OR; Symmetry OR; Asymmetry OR; Swing OR; Dual task* OR; Single support* OR; Double support* OR; Double limb OR; Stance OR; Ambul*
**Neuroimaging**	**MESH headings**: Neuroimaging; Tomography; Electroencephalography; Evoked potentials; Spectroscopy, Near-Infrared **Key words:** SPECT; MRI; Magnetic resonance*; Neural network*; Resting state; DTI; Connectivity; fMRI; sMRI; PET; CT; VBM; Voxel based morphometry; EEG; fNIRS; FLAIR; Fluid-attenuated inversion recovery; DaTSCAN; Dopaminergic imaging	**MESH headings:** Spectroscopy; Neural networks; Brain Connectivity; Neuroimaging; Tomography; Electroencephalography; Evoked potentials **Key words:** fMRI; sMRI; MRI; Magnetic resonance*; Resting state; DTI; Functional Neuroimaging; Brain Mapping; Connectome; SPECT; PET; CT; VBM; Voxel based morphometry; EEG; fNIRS; functional near infra-red spectroscopy; FLAIR; Fluid-attenuated inversion recovery; DaTSCAN; Dopaminergic imaging	**Key word search:** Magnetic Resonance* OR; MRI OR; Neural network* OR; Resting state OR; Diffusion tensor imaging OR; DTI OR; Connectivity OR; fMRI OR; sMRI OR; Neuroimaging OR; Neuroradiography OR; Functional Neuroimaging OR; Brain Mapping OR; Connectome OR; Tomography OR; Computed tomography OR; Single Photon Emission Computed Tomography OR; Positron Emission Tomography OR; SPECT OR; PET OR; CT OR; Susceptibility weighted imaging OR; Optical tomography OR; Diffuse optical tomography OR; VBM OR; Voxel based morphometry OR; EEG OR; Electroencephalography OR; Evoked potential OR; Event related potential OR; fNIRS OR; functional near infra-red OR; spectroscopy OR; FLAIR OR; Fluid-attenuated inversion recovery OR; DaTSCAN OR; Dopaminergic imaging OR; Arterial spin labelling
**Old age**	“All aged” limit set	“Aged” limit set	**Key word search:** Old* OR; Aged OR; Aging OR; Elderly OR; Senior*

### 2.2 Inclusion and exclusion criteria

Articles were included if they assessed gait in healthy older adults under single task conditions, and used at least one brain imaging technique. As we were interested in looking specifically at quantitative gait characteristics, complex paradigms which involved standing or turning, such as the Timed Up and Go task, were excluded, as were protocols which made use of imagined gait. Additionally, we did not consider walks performed under dual-task conditions (which involve a secondary task being completed whilst walking), as the methodologies of these dual-task paradigms greatly vary and may cause different impairments in gait (Beurskens et al., 2014; Doi et al., 2017), the details of which are not well explored and are beyond the scope of this review. Articles involving animal models, case studies, intervention studies, clinical trials or only nerve or brain stimulation were excluded, as were those that only assessed falls, freezing of gait or general physical activity.

### 2.3 Data extraction

Once duplicates were removed, one reviewer (J.W.) screened the titles from the initial search, and two reviewers (J.W. and L.A.) independently screened the abstracts to identify potential articles. The full-text of articles was retrieved if reviewers were unable to determine the eligibility of the study from the title and abstract alone. All full-length articles were assessed by three reviewers (J.W., L.A., and R.M.A.). Data extraction forms were completed, which included information about population characteristics, whether the study was cross-sectional or longitudinal, the study inclusion and exclusion criteria, the gait analysis technique and variables measured, the imaging technique and variables measured, the statistical tools used, and the main study findings. A quality assessment was conducted separately by two reviewers (J.W and R.M.A) and overall quality scores were determined for each study (see Supplementary Table 1).

## 3 Results

### 3.1 Search yield

**Fig. 2:**
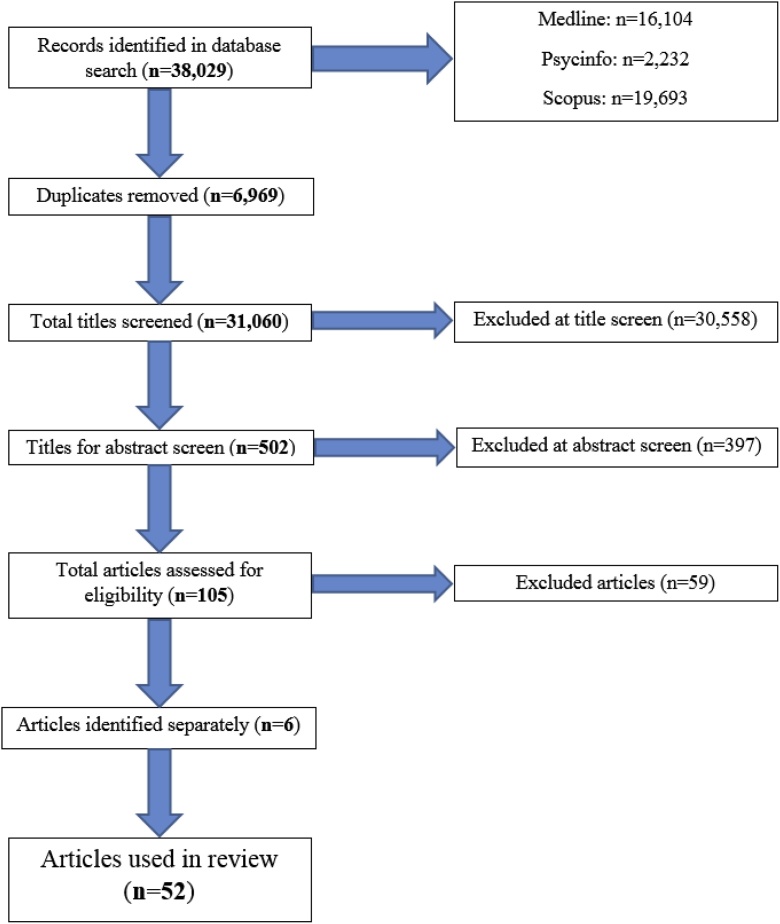
A prisma diagram demonstrating the search yield for the structured review.

The search yield is shown in Fig. 2. The search, completed on 04.04.2018, generated a total of 38,029 studies after search limits were applied. Once duplicates were removed, a total of 31,060 studies were yielded from the search. After the initial title screen, 502 studies were identified as being of interest; 105 studies were then eligible for data extraction after abstract screening. 59 studies were excluded during data extraction, as no single task gait was completed (n = 4); only the timed up and go task was completed (n = 2); the gait assessment involved a turn (n = 3); the gait measurement tool was not described (n = 1); derived gait measures were unsuitable (n = 21); access to the paper was unavailable (n = 2); the image analysis undertaken only involved brain or nerve stimulation (n = 1); the age range investigated was inappropriate (n = 3); the type of article was unsuitable (n = 2); only results relating to disease cohorts were presented (n = 11); only results representing group comparisons were presented (n = 1); the direction of association between the variables was not specified (n = 1), or there was no direct link between the two variables of interest (n = 7). Six additional studies have been identified outside of the search strategy since the search closed. Therefore, 52 studies are included in this review. Publication dates range from 1997 to 2018.

### 3.2 Brain imaging and gait assessments - methodological comparisons

Six imaging modalities were described in the studies included in this review. The majority of studies used structural magnetic resonance imaging (MRI) for brain imaging; 33 studies used structural MRI as the only imaging technique (Rosano et al., 2005a, b; Wolfson et al., 2005; Rosano et al., 2007a, b; Baezner et al., 2008; Rosano et al., 2008; Nadkarni et al., 2009; Novak et al., 2009; Soumaré et al., 2009; Murray et al., 2010; de Laat et al., 2011b; Frederiksen et al., 2011; Ryberg et al., 2011; Sorond et al., 2011; Choi et al., 2012; de Laat et al., 2012; Dumurgier et al., 2012; Manor et al., 2012; Moscufo et al., 2012; Callisaya et al., 2013; Willey et al., 2013; Wolfson et al., 2013; Annweiler et al., 2014; Beauchet et al., 2014; Bolandzadeh et al., 2014; Callisaya et al., 2014; Nadkarni et al., 2014; Beauchet et al., 2015; Ezzati et al., 2015; Rosario et al., 2016; Stijntjes et al., 2016; Beauchet et al., 2017), five studies used both structural MRI and diffusion tensor imaging (DTI) techniques (Della Nave et al., 2007; de Laat et al., 2011a, c; Rosso et al., 2014; van der Holst et al., 2018), and one study combined the use of structural MRI with magnetic resonance spectroscopy (MRS) (Zimmerman et al., 2009). Four studies used imaging parameters derived from DTI images only (Bruijn et al., 2014; Fling et al., 2016; Verlinden et al., 2016; Fling et al., 2018), one study used functional MRI (Yuan et al., 2015), positron emission tomography (PET) was used in seven studies (Shimada et al., 2013; Sakurai et al., 2014; del Campo et al., 2016; Nadkarni et al., 2017; Sakurai et al., 2017; Tian et al., 2017b; Wennberg et al., 2017a), and functional near infrared spectroscopy (fNIRS) was used in only one study (Holtzer et al., 2015).

Eight different quantitative gait measurement techniques were reported. Twenty-seven studies reported the use of gait walkway systems (Rosano et al., 2005a, b; Rosano et al., 2008; Nadkarni et al., 2009; Zimmerman et al., 2009; Murray et al., 2010; de Laat et al., 2011a, b, c; Choi et al., 2012; de Laat et al., 2012; Callisaya et al., 2013; Annweiler et al., 2014; Bolandzadeh et al., 2014; Callisaya et al., 2014; Nadkarni et al., 2014; Rosso et al., 2014; Beauchet et al., 2015; Ezzati et al., 2015; Holtzer et al., 2015; Yuan et al., 2015; Rosario et al., 2016; Verlinden et al., 2016; Beauchet et al., 2017; Wennberg et al., 2017a; Fling et al., 2018; van der Holst et al., 2018), one study used both a gait walkway and inertial sensors to measure different gait characteristics (Fling et al., 2016), two studies utilised two photoelectric cells connected to a chronometer (Soumaré et al., 2009; Dumurgier et al., 2012), footswitches were used in two studies (Manor et al., 2012; Beauchet et al., 2014), an accelerometer was described in one study (Stijntjes et al., 2016), one study made use of a treadmill (Shimada et al., 2013), one study described the use of reflective markers with a motion analysis system during treadmill walking (Bruijn et al., 2014) and 17 studies derived gait characteristics from timed walks (Rosano et al., 2005b; Wolfson et al., 2005; Della Nave et al., 2007; Rosano et al., 2007a; Baezner et al., 2008; Novak et al., 2009; Frederiksen et al., 2011; Ryberg et al., 2011; Sorond et al., 2011; Moscufo et al., 2012; Willey et al., 2013; Wolfson et al., 2013; Sakurai et al., 2014; del Campo et al. (2016); Nadkarni et al., 2017; Sakurai et al., 2017; Tian et al., 2017b). Gait characteristics relating to intraindividual variability were derived either as the standard deviation of the variability within the original measurement (Verlinden et al., 2016; Zimmerman et al., 2009) or as a coefficient of variance of the measurement (Annweiler et al., 2014; Beauchet et al., 2014, 2015, 2017; de Laat et al., 2011c; Manor et al., 2012; Rosano et al., 2007b; Rosso et al., 2014; Shimada et al., 2013; Wennberg et al., 2017a).

### 3.3 Associations between brain structure and function and gait characteristics

Associations between quantitative gait characteristics and structural and functional imaging parameters were explored. In order to examine the wide variety of gait characteristics assessed, all gait characteristics included within studies were mapped onto the five domains of gait outlined in Lord et al’s gait model (Lord et al., 2013). Studies assessing either steps or strides were considered and grouped together for the purposes of this review; definitions of the gait terms used have been described in supplementary Table 2. Imaging parameters were also grouped by the imaging technique used for an effective interpretation of results. Imaging parameters derived from structural MRI were divided in to two groups, those assessing brain volumes and those investigating white matter changes that are common in ageing. Where possible, the specific brain regions in which associations were made have been reported, as well as any covariates included in statistical analyses.

**Fig. 3:**
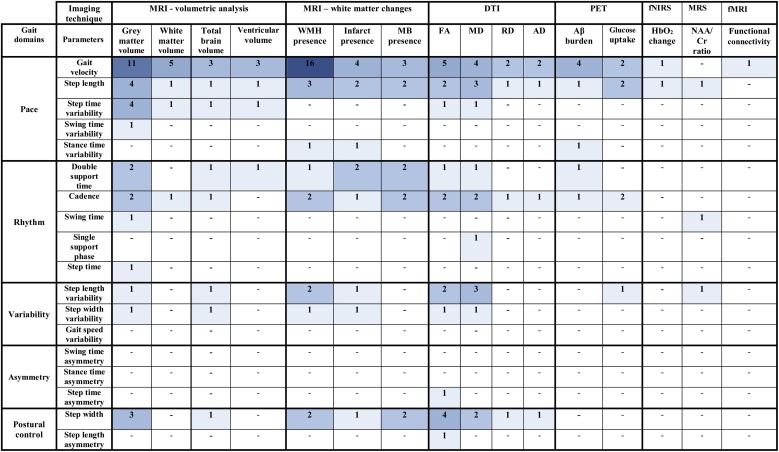
Heat map of the cross-sectional studies assessing each imaging and gait parameter.

47 studies described cross-sectional associations between gait and brain imaging parameters (Rosano et al., 2005a, b; Della Nave et al., 2007; Rosano et al., 2007a, b; Baezner et al., 2008; Rosano et al., 2008; Nadkarni et al., 2009; Novak et al., 2009; Soumaré et al., 2009; Zimmerman et al., 2009; Murray et al., 2010; de Laat et al., 2011a, b, c; Sorond et al., 2011; Choi et al., 2012; de Laat et al., 2012; Dumurgier et al., 2012; Manor et al., 2012; Moscufo et al., 2012; Shimada et al., 2013; Willey et al., 2013; Wolfson et al., 2013; Annweiler et al., 2014; Beauchet et al., 2014; Bolandzadeh et al., 2014; Bruijn et al., 2014; Callisaya et al., 2014; Nadkarni et al., 2014; Rosso et al., 2014; Sakurai et al., 2014; Beauchet et al., 2015; Ezzati et al., 2015; Holtzer et al., 2015; Yuan et al., 2015; del Campo et al. (2016); Fling et al., 2016; Rosario et al., 2016; Stijntjes et al., 2016; Verlinden et al., 2016; Beauchet et al., 2017; Nadkarni et al., 2017; Sakurai et al., 2017; Tian et al., 2017b; Wennberg et al., 2017a; Fling et al., 2018). Fig. 3 summarises the number of studies comparing each gait characteristic to each imaging parameter; detailed descriptions of each cross-sectional study are included in Table 2.

**Table 2:** Descriptive information of all cross-sectional studies. Participant ages have been reported as mean ± standard deviation unless stated otherwise.

Study	Participant characteristics	Gait variables measured in pace domain	Gait analysis tool	Imaging parameters measured	Imaging modality and analytical technique utilised	Main Findings	Covariates	Effect of covariates on findings
1. Annweiler et al. (2014)	Older Adults (n = 115), 43.5% F, age 70.4 ± 4.4	Stride time variability (%CV)	GAITRite (9.72 m)	Ventricular sub-volumes (total, temporal horns and ventricular body volumes)	MRI: Brain Ventricle Quantification Suite (BVQ)	The highest tertile of stride time variability had larger temporal horns than the lowest and intermediate tertiles, and larger middle portions of ventricular bodies than the intermediate, but not lowest, tertile	CIRS-G, lower limb proprioception, visual acuity, Go-NoGo, MMSE, GDS, muscle strength, age, gender, BMI, gait speed, vascular burden in brain, psychoactive drugs	After adjusting for covariates, posterior body volume no longer associated with stride time variability
2. Baezner et al. (2008)	Older Adults (n = 639), 53.9% F, age range 65-84	Gait velocity (ms^−1^)	Timed walk (8 m; fastest of two trials)	Age-related white matter changes (ARWMC), ranked as mild, moderate or severe, using a modified version of the Fazekas visual scale	MRI: Fluid-attenuated inversion recovery (FLAIR)	Slower gait velocity associated with more severe global age-related white matter changes, in mild vs moderate and mild vs severe conditions	age, sex, peripheral vascular disease, diabetes	When covariates included in analyses, associations remained in the mild vs. severe condition, but not in the mild vs. moderate condition
3. Beauchet et al. (2014)	Older Adults (n = 71), 59.7% F, age 69 ± 0.8	Stride time variability (%CV)	SMTEC footswitches (20 m, comfortable walking pace)	Regional grey and white matter volumes (Fazekas visual scale of ARWMC was assessed for exclusion criteria)	MRI: Voxel based morphometry (VBM)	Increased stride time variability associated with reduced grey matter volume in the right parietal lobe. There was no association between stride time variability and white matter volume, perhaps as partipants with extreme ARWMC were excluded	age, gender, BMI, total brain matter	All associations were made with inclusion of covariates
4. Beauchet et al. (2015)	Older Adults (n = 47), 49% F, age 69.7 ± 3.6	Stride time variability (%CV), Swing time variability (%CV), Stride time (ms), Swing time (ms), Stride width variability (%CV), Stride width (cm)	GAITRite Gold (9.72 m)	Hippocampal volume: absolute hippocampal volume mm^3^ and ratio of absolute hippocampal volume mm^3^ / total brain volume mm^3^	MRI: Freesurfer morphometric procedures	Increased stride time variability associated with a larger hippocampal volume. There were no associations between swing time variability, stride time, swing time stride width variability or stride width and hippocampal volume.	age, gender, BMI, daily drugs taken, falls within last year, gait velocity, total white matter abnormalities, MMSE	When covariates were *not* adjusted for, associations were not significant
5. Beauchet et al. (2017)	Older Adults (n = 77), 45.5% F, age 69.8 ± 3.5	Gait speed (ms^−1^), Stride time variability (%CV)	GAITRite Gold (9.72 m, comfortable walking pace)	Hippocampal and somatosensory cortex volumes	MRI: Freesurfer morphometric procedures	No associations were made between gait speed or stride time variability and hippocampal or somatosensory cortex volumes	age, sex, body mass index, comorbidities, use of psychoactive drugs, far-distance visual acuity, lower-limb proprioception, depressive symptoms and cognitive scores (Mini-Mental State Examination, Frontal Assessment Battery)	Inclusion of all covariates in analyses had no significant effect on associations
6. Bolandzadeh et al. (2014)	Older Adults (n = 253), 58.0% F, age 82.7 ± 2.7	Gait speed (ms^−1^)	GaitMat II (3, 4 and 6 m walks)	White matter hyperintensity volume. Selected regions of interest: global brain volume, right anterior thalamic radiation and frontal corpus callosum volume	MRI: Automated labelling pathway (ALP)	Slower gait speed associated with higher global white matter hyperintensity volume, and most strongly with white matter hyperintensities in the right anterior thalamic radiation and frontal corpus callosum	age, sex, BMI, quadriceps strength, education, standing height, chronic pain, Prevelant hypertension, 3MS (global cognition), DSST (executive function)	Associations reduced when 3MS or DSST were included in analyses – when adjusted, associations between gait speed and frontal corpus callosum lose significance.
7. Bruijn et al. (2014)	Older Adults (n = 25), 40.0% F, age 70.9	Step width (m)	Vicon MX markers (300 m at fixed pace on treadmill)	Fractional anisotropy	DTI: Tract-Based Spatial Statistics (TBSS)	A narrower step width associated with reduced fractional anisotropy, in the left corticospinal tract, left anterior thalamic radiation and left longitudinal fasciculus.	age	This association was found in older adults; younger adults also demonstrated the relationship, but to a lesser extent
8. Callisaya et al. (2014)	Older Adults (n = 305), 45.9% F, age 71.4 ± 6.9	Gait speed(cm/s), Step length (cm), Cadence (steps per minute)	GAITRite (4.6 m)	Total grey and normal appearing white matter volumes	MRI: Voxel based morphometry (VBM)	Slower gait speed and shorter step length associated with reduced total grey matter volume, and with reduced grey matter within regions in all cortices (frontal, cingulate, insula, temporal, parietal, occipital) as well as the cerebellum, insula, parahippocampus, thalamus, caudate nucleus, putamen and claustrum. No associations were made with gait speed or step length and normal appearing white matter, or between cadence and total grey or white matter volumes	1. Age, sex, height 2. Vascular risk factors (weight, blood pressure, physical activity or self-reported vascular medical history), medication use 3. WMH volume, total grey or white matter volume (as appropriate)	All associations were made with inclusion of covariates 1 (age, sex and height.) When adding (2) there were no alterations. When adding (3) associations with grey matter were minimally altered – removing associations with gait speed and the right precuneus, left caudate nucleus, left thalamus, left inferior occipital and left inferior temporal lobes, right superior parietal and right inferior occipital lobes, right precuneus, right insula and left anterior cingulate areas. Associations with step length only minimally altered.
9. Choi et al. (2012)	Older Adults (n = 377), age and gender characteristics grouped by presence of infarcts and microbleeds	Gait speed (cm/s), Step length (cm), Cadence (steps per minute), Double support time (s), Step width (cm)	GAITRite (4.6 m)	Silent infarcts and cerebral microbleed presence	MRI: Fluid-attenuated inversion recovery (FLAIR)	Slower gait speed, smaller step length and wider steps associated with increased global silent infarct presence, but not global microbleed presence. Longer double support time associated with increased presence of silent infarcts and microbleeds. Reduced cadence associated with increased microbleed, but not silent infarct, presence.	age, sex, total intracranial volume, white matter lesion volume, microbleeds or silent infarcts as appropriate	Inclusion of all covariates in analyses caused associations to no longer be statistically significant
10. De Laat et al. (2011a)	Older Adults (n = 429), 45.2% F, age 65.2 ± 8.9	Gait velocity (ms^−1^), Stride length (m), Cadence (steps per minute), Stride width (cm)	GAITRite (5.6 m)	White matter lesion (WML) presence, fractional anisotropy (FA), mean diffusivity (MD)	MRI: Fluid-attenuated inversion recovery (FLAIR) DTI: Tract-Based Spatial Statistics (TBSS)	Slower gait velocity, shorter stride length and wider stride width associated with WML presence in the right centrum semiovale. Shorter stride length also associated with increased WML presence in periventricular frontal lobes. No significant associations were identified between WML presence and cadence. Slower gait velocity, shorter stride length and wider strides also associated with reduced FA and increased MD in most regions, including normal-appearing white matter and white matter lesion regions. Less cadence associated with reduced FA and increased MD in few voxels.	age, sex, height, WML total volume, number of lacunar infarcts or total brain volume	Most associations between gait velocity or stride length and white matter integrity parameters were no longer significant when white matter lesions and lacunar infarcts were included as covariates. Associations that remained were those between fractional anisotropy and stride length in the genu and splenium of the corpus callosum, and between mean diffusivity and stride length in the body of the corpus callosum
11. De Laat et al. (2011bd)	Older Adults (n = 485), 43.1% F, age 65.6 ± 8.8	Gait velocity (ms^−1^), Stride length (m), Cadence (steps per minute), Double support percentage (% of gait cycle), Stride width (cm)	GAITRite (5.6 m)	Microbleeds: number and location	MRI: gradient-echo T2*-weighted sequences	Slower gait velocity and shorter stride length associated with an increased number of microbleeds, both globally and in the temporal lobe. Stride length was also associated with the number of microbleeds in the frontal lobes and basal ganglia. Cadence was not associated with the number of microbleeds. Increased double-support percentage and wider strides associated with an increased number of microbleeds (regional burden was not considered for these gait characteristics).	age, sex, height, total brain volume, number of territorial infarcts, WML total volume, number of lacunar infarcts	Inclusion of all covariates in analyses did not significantly alter associations with stride length, but caused associations with gait velocity to no longer be significant
12. De Laat et al. (2011c)	Older Adults (n = 484), 43.4% F, age 65.6 ± 8.9	Gait velocity (ms^−1^), Stride length (m), Stride time variability (%CV), Cadence (steps per minute), Double support percentage (% of gait cycle), Stride length variability (%CV), Stride width variability (%CV), Stride width (cm)	GAITRite (5.6 m)	Total brain volume, fractional anisotropy (FA), mean diffusivity (MD)	MRI: SPM unified segmentation routines DTI: SPM5	Slower gait velocity, shorter stride length, increased stride time variability, less cadence, greater double support percentage, increased stride length variability and larger stride width associated with a reduced total brain volume. Reduced FA in normal appearing white matter associated with slower gait velocity, shorter step length, reduced cadence and larger stride width. Increased MD in both normal appearing white matter and white matter lesions was associated with slower gait velocity, shorter step length, reduced cadence, greater double support percentage, and larger stride width. Increased MD was associated with increased stride length variability in normal appearing white matter only, and with reduced stride width variability in white matter lesions only. The associations between FA and gait velocity were significant within periventricular frontal, occipital and temporal regions; associations between gait velocity and MD were additionally significant in subcortical frontal and parietal regions.	age, sex, height, total brain volume (where appropriate), white matter lesion total volume, number of lacunar infarcts	Including WML volume and number of lacunar infarcts as covariates caused relationship with fractional anisotropy, but not mean diffusion, disappear. Associations made with total brain volume were made whilst including all covariates in analyses
13. de Laat et al. (2012)	Older Adults (n = 415), 46.3% F, age 65.1 ± 8.8	Gait velocity (ms^−1^), Cadence (steps per minute), Stride length (m), Stride width (cm)	GAITRite (5.6 m)	Cortical thickness	MRI: Civet Pipeline	Slower gait velocity, shorter stride length, reduced cadence and larger stride width associated with decreased cortical thickness. Associations with gait velocity were made in areas involving most of the cortex, associations with stride length were made in most frontal regions except for the bilateral motor cortices, visual areas and anterior and posterior cingulate areas, associations with cadence were mostly within the left cingulate, bilateral visual areas and the left fusiform gyrus and stride width associations were made within the orbitofrontal and ventrolateral prefrontal cortex, the cortical areas adjacent to the posterior insula, inferior temporal gyrus, left fusiform gyrus and dorsal anterior cingulate cortex	age, sex, height, WML total volume, number of lacunar infarcts	Associations weakened after the inclusion of WML total volume as a covariate, but mostly remained significant
14. del Campo et al. (2016)	Older Adults (n = 128), 60.2% F, age 76.1 ± 4.6	Gait speed (ms^−1^)	Timed walk (4 m; fastest of two trials)	Amyloid beta burden assessed through standard uptake value ratios (SUVR’s)	PET: [18 F]	Slower gait speed associated with increased amyloid beta burden within the putamen, occipital cortex, precuneus, and anterior cingulate	age, sex, education, BMI, APOE genotype, days since baseline at PET, time between gait and PET assessments, regional 18 F standard uptake value ratios (as appropriate)	Association was made with inclusion of covariates
15. Della Nave et al. (2007)	Older Adults (n = 36), 58.0% F, age 77 ± 4.5	Gait velocity (ms^−1^)	Timed walk (4 m)	Leukoaraiosis through the Fazekas scale (LA), total brain volume, grey matter volume, white matter volume, fractional anisotropy (FA), mean diffusivity (MD)	MRI: SIENAX DTI: Philips in-house software	Results were unclear; It is interpreted that gait velocity was not associated with any of the imaging parameters investigated	not reported	Unknown
16. Dumurgier et al. (2012)	Older Adults (n = 1623), 60.5% F, age 73.3 ± 4.1	Walking speed (ms^−1^)	Chronometer connected to 2 photoelectric cells (6 m; fast walking condition)	Regional grey matter volume	MRI: Voxel based morphometry (VBM)	Slower walking speed associated with reduced grey matter volume; regions that reached statistical significance were the basal ganglia and caudate nucleus.	age, sex, BMI, education, WML volume, silent infarcts, total intracranial volume, MMSE or TMT-A, depression, hypertension, diabetes, hypercholesterolemia, smoking. P values were Bonferroni corrected	Adjusting for cognitive test scores caused associations with frontal and parietal lobes to no longer be significant
17. Ezzati et al. (2015)	Older Adults (n = 112) 59.8% F, age 79.3 ± 5.0	Gait velocity (cm/s)	GAITRite (4.6 m; normal pace, two trials)	Cortical grey matter volume, cerebral total white matter volume, hippocampal volume, ventricular volume	MRI: Freesurfer standard segmentation procedures and Freesurfer morphometric procedures	In unadjusted models, slower gait velocity was associated with reduced grey matter, white matter and hippocampal volumes. No association was made with gait velocity and ventricular volume	age, gender, education, total intracranial volume, memory performance. p-values were corrected for type one error	Associations with white matter and hippocampal volumes were not significant after adjusting for covariates
18. Fling et al. (2016)	Older Adults (n = 10), 60% F, average age 75 (62-84 age range)	Gait velocity (ms^−1^), Stride width (cm)	GAITRite (8 m)	Fractional anisotropy, mean diffusivity	DTI: Interhemispheric Callosal Tractography	No associations were made between gait velocity or stride width and fractional anisotropy or mean diffusivity.	not reported	Unknown
19. Fling et al. (2018)	Older Adults (n = 20), 65% F, age 71.4 ± 8.1	Step length asymmetry (%), Step time asymmetry (%)	GAITRite (8 m)	Fractional anisotropy	DTI: Interhemispheric Callosal Tractography. Regions investigated: pre-supplementary motor area, supplementary motor area, primary motor and primary somatosensory	No associations were made between either step length asymmetry or step time asymmetry and fractional anisotropy in any of the regions of interest.	not reported	Unknown
20. Holtzer et al. (2015)	Older Adults (n = 348), 59.0% F, age 76.8 ± 6.8	Stride velocity (cm/s), Stride length (cm)	Zeno walkway (14 foot)	Activation strength (due to oxyHb levels)	fNIRS: 16-channel	No associations were made between gait velocity or stride length and activation strength of the prefrontal cortex	disease comorbidity score, RBANS (cognition), age, sex, education	Association was made with inclusion of covariates
21. Manor et al. (2012)	Older Adults (n = 89), 46.5%F, age 65.3 ± 8.2	Walking speed (ms^−1^), Stride duration variability (%CV), Double support (% of stride time)	Timed 12 minute walk using Mega Electronics heel and Toe footswitches (75 m course)	Global and regional grey matter volume. Specific regions of interest: right and left precentral gyri, basal ganglia and cerebellum, post central gyri and dorsolateral prefrontal cortex.	MRI: SPM segmentation routines	No associations were made between walking speed, stride duration variability or double support time and global, cerebellar dorsolateral prefrontal cortex or basal ganglia grey matter volumes.	age, sex, body mass	Associations were made with inclusion of covariates
22. Moscufo et al. (2012)	Older Adults (n = 77), 60% F, age 84 ± 3.9	Walk speed (ms^−1^) (max velocity and usual velocity)	SPPB (2.5 m)	WMH volume. Selected regions of interest – corpus collosum, corona radiate, superior longitudinal fasiculus.	MRI: Fluid-attenuated inversion recovery (FLAIR)	At two separate time points, maximum and usual walk speeds associated with WMH burden in the splenium of the corpus callosum. At the earlier time point, both walking speeds were additionally associated with WMH burden in the corona radiata; at the later time point, both walking speeds were additionally associated with WMH burden in the body of the corpus callosum	age, gender, mini-mental state score (MMSE), and body mass index (BMI)	Inclusion of all covariates in analyses had no effect on associations
23. Murray et al. (2010)	Older Adults (n = 148), 56.1% F, average age 79 (73-91 age range)	Gait velocity (cm/s), Stride length (cm)	GAITRite (4.9 m)	WMH volume	MRI: Fluid-attenuated inversion recovery (FLAIR)	Slower gait velocity and shorter stride length associated with an increased number of white matter hyperintensities in all regions (total, periventricular, subcortical, frontal temporal, parietal and occipital).	not reported	Unknown
24. Nadkarni et al. (2009)	Older Adults (n = 33), 47% F, age 73 ± 8	Gait velocity (cm/s), Stride length (cm), Cadence (steps per minute), Step width (cm)	GAITRite (12 foot)	WMH severity	MRI: Age related white matter change rating scale; results were reported through groups split by white matter burden	Slower gait velocity associated with increased severity of total white matter hyperintensities, as well as WMH severity in frontal and basal ganglia regions. No associations were made between stride length or cadence and WMH. Narrower steps associated with increased WMH severity in basal ganglia regions.	age, UPDRS, MMSE, dementia rating scale	Not specified
25. Nadkarni et al. (2014)	Older Adults (n = 231), 58.4% F, age 82.9 ± 2.7	Gait speed (ms^−1^)	GaitMat II (4 m)	Cerebellar grey matter volume	MRI: hidden Markov random field (HMRF) model	Slower gait speed associated with reduced total cerebellar volume, as well as cognitive and sensorimotor cerebellar regions.	age, gender, WMH, atrophy, DSST	After adjusting for DSST, the association between larger cerebellar volume and faster gait speed was no longer statistically significant
26. Nadkarni et al. (2017)	Older Adults (n = 183), 41.5% F, age 85.5 ± 3	Gait speed (ms^−1^)	Timed walk (4.57 m)	Amyloid beta burden through standard uptake volume ratios (SUVRs)	PET: [11C]PiB	Slower gait speed associated with global increased amyloid beta burden, and amyloid beta burden in anterior caudate and putamen, lateral temporal cortex, precuneus cortex and sensory-motor cortex	age, sex, race, education, weight, hypertension, coronary heart disease, stroke, cortical atrophy, SVD, MMSE, APOE ε4	When MMSE was included as a covariate, associations lessened but persisted. When APOE ε4 was included as a covariate, associations were no longer significant
27. Novak et al. (2009)	Older Adults (n = 76), 52.6% F, age 64.7 ± 7.2	Gait speed (ms^−1^)	Timed walk (12 minutes)	Frontal grey and white matter volume, WMH volume	MRI: Fluid-attenuated inversion recovery (FLAIR)	Slower gait speed associated with reduced frontal grey and white matter volumes. No association was made between gait speed and white matter hyperintensity volume.	age, BMI, posturographic measures	Associations were made with inclusion of covariates
28. Rosano et al. (2005a)	Older Adults (n = 321), 60.7% F, age 78.3	Gait speed (ms^−1^), Stride length (m), Double support time (s)	GaitMat II (4 m)	WMH volume, infarcts, ventricular enlargement	MRI: comparison to an atlas of predefined visual standards	Slower gait speed associated with an increase in the number of subcortical and basal ganglia infarcts and WMH. Stride length did not associate with the number of WMH, but did associate with an increase in the number of subcortical and basal ganglia infarcts. Longer double support time associated with an increase in total WMH, but not infarct number. No gait characteristic was associated with ventricular enlargement.	age, sex, race, CVD risk factors	Adjusting for covariates had little effect on relationship identified
29. Rosano et al. (2005b)	Older Adults (n = 2450), 57% F, age 74.4 ± 4.7	Gait speed (ms^−1^)	Timed walk (15 foot)	Ventricular enlargement, WMH volume, infarcts	MRI: comparison to an atlas of predefined visual standards	Slower gait speed associated with increased ventricular size, an increase in the severity of WMHs and infarct presence.	age, sex, race, education, cardiovascular risk factors, cardiovascular disease, 3 ms, incident stroke, incident dementia	Adjusting for covariates had little effect on relationship identified
30. Rosano et al. (2007a)	Older Adults (n = 327), 56.5% F, age 78.2 ± 3.9	Gait speed (ms^−1^)	Timed walk (15 foot)	Regional grey matter volume	MRI: Automated labelling pathway (ALP)	Slower gait speed associated with a reduction in grey matter volume in the precuneus, left cerebellum and prefrontal regions.	gender, education, BMI, head size, osteoarthritis, peripheral arterial disease, WMH, infarcts, ventricular enlargement	Adjusting for covariates had little effect on relationships identified
31. Rosano et al. (2007b)	Older Adults (n = 331), % F unknown, age 78.3 ± 4.0	Stance time variability (CV%), Step length variability (%CV), Step width variability (%CV)	GaitMat II (4 m)	Total and basal ganglia infarcts and WMH severity	MRI: comparison to an atlas of predefined visual standards	Increased stance time variability associated with WMH severity, but not the number of total or basal ganglia infarcts. Increased step length variability associated with WMH severity and the number of total and basal ganglia infarcts. Step width variability did not correlate with any imaging parameter	age, gender, 3MSE, CV diseases, UPDRS, CES-D, BMI, hip/knee pain, joint arthritis	Associations were made with inclusion of covariates
32. Rosano et al. (2008)	Older Adults (n = 220), 63.2% F, age 78 ± 3.9	Step length (m), Double support time (s), Step width (m)	GaitMat II (4 m)	Regional grey matter volume	MRI: Region of Interest (ROI)	Shorter step length, longer double support time amd wider steps associated with reduced of grey matter volume. Regionally, step length associated with the dorsolateral prefrontal cortex, left supplementary motor cortex, right parietal lobules, motor cortex and sensorimotor cortex. Double support time associated the dorsolateral prefrontal cortex, right parietal lobules, right motor cortex and sensorimotor cortex. Wider steps associated the right dorsolateral prefrontal cortex pallidum and inferior parietal lobe.	age, gender, total brain volume, BMI, arthritis, sensory impairment, ankle arm ratio, infarct, WMH, stroke, 3MSE, DSST, CES-D, dementia	Adjusting for covariates had little effect on step length relationships identified; only the association between left motor cortex and step length was attenuated
33. Rosario et al. (2016)	Older Adults (n = 265), 57% F, age 82.9 ± 2.7	Gait speed (ms^−1^)	GaitMat II (4 m)	WMH volume	MRI: Fluid-attenuated inversion recovery (FLAIR) (DTI imaging also completed, but for use as a covarying factor only)	Slower gait speed associated with an increased number of white matter hyperintensities, with stronger associations in the corona radiata, superior longitudinal and fronto-occipital fasciculus, uncinate fasciculus, anterior limb of internal capsule, posterior limb of internal capsule and left retrolenticular part of internal capsule, right posterior thalamic radiation, external capsule and corpus callosum. Associations were made in many regions in participants with low fractional anisotropy, but in few regions in participants with high fractional anisotropy.	age, sex, fractional anisotropy, muscle strength, body mass index, diabetes, hypertension, stroke	Associations held after adjustment for age and sex, and after adjustment for fractional anisotropy.
34. Rosso et al. (2014)	Older Adults (n = 265), 57.4% F, age 82.9 ± 2.7	Step length variability (%CV)	GaitMat II (4 m)	Grey matter atrophy, WMH volume, fractional anisotropy, mean diffusivity	MRI: Automated segmentation (FAST) and Fluid-attenuated inversion recovery (FLAIR) DTI	Increased step length variability associated with increased mean diffusivity within the hippocampus and anterior cingulate cortex	age, gender, obesity, diabetes, muscle strength, gait speed, CES-D, DSST	Associations were made with inclusion of covariates.
35. Sakurai et al. (2014)	Older Adults (n = 182), 100% F, age 69.4 ± 6.6	Gait speed (m/minute), Step length (cm), Step frequency (steps per second)	Timed walk (unspecified) (5 m) (comfortable speed and max speed)	Normalised regional cerebral metabolic rates of glucose uptake	PET: 18 F	Slower gait speed and lower step frequency during walking at maximum speed associated with a reduction in metabolic rates of glucose uptake in the prefrontal, posterior cingulate, and parietal cortices. There was no association between step length and metabolic rates of glucose uptake when assessed at maximum gait speed, or with any gait characteristics assessed at comfortable pace.	age, BMI, education, hypertension, cardiac disease, diabetes mellitus, blood pressure, days between assessment	Associations were made with inclusion of covariates
36. Sakurai et al. (2017)	Older Adults (n = 149), 100% F, age 70.2 ± 6.2	Gait speed (ms^−1^), Step length (cm), Step frequency (steps per second)	Timed walk (unspecified) (5 m) (comfortable and fast walk)	Normalised regional cerebral metabolic rates of glucose	PET: 18 F	During fast walking, slower gait speed and lower step frequency were associated with a reduction in metabolic rates of glucose in the posterior cingulate, occipital, parietal and primary sensorimotor cortices. There was no association between step length during fast walking and metabolic rates of glucose. No associations were made when walking parameters assessed at a comfortable pace	age, education, comorbidities, blood glucose level, BMI,	Associations were made with inclusion of covariates
37. Shimada et al. (2013)	Older Adults (n = 24), 100% F, age 78.0 ± 2.3	Step length variability (%CV), participants were split in to low or high step length variability groups	Treadmill walk with infrared step counter (25 minutes at 2 km/h, so approx. 833 m)	Metabolic rates of glucose	PET: 18 F	The low step length variability (LSV) group had relatively increased glucose uptake in the primary sensorimotor area in comparison to the high step length variability (HSV) group. The HSV group had comparatively decreased uptake in the middle and superior temporal gyrus and hippocampus in relation to the LSV group	not reported	Unknown
38. Sorond et al. (2011)	Older Adults (n = 42), 54.8% F, age 76 ± 5 (fast gait), 82 ± 6 (slow gait)	Gait speed (ms^−1^)	Timed walk (4 m) (fastest of 2 trials)	WMH volume, brain parenchymal volume	MRI: Fluid-attenuated inversion recovery (FLAIR)	Slower gait speed associated with a higher burden in the total number of white matter hyperintensities. No significant associations were made between gait speed and brain parenchymal volume, although there was a trend towards this relationship	not reported	Unknown
39. Soumaré et al. (2009)	Older Adults (n = 1702), 60.6% F, age 72.4 ± 4.1	Walking speed (ms^−1^)	Chronometer connected to 2 photoelectric cells (6 m) (maximum speed)	WMH volume	MRI: Fully automated software	Slow walking speed was associated with the highest number of total, periventricular and deep WMH	age, gender, education, brain white matter volume, BMI, homocysteine level, psychotropic drugs, hypertension, physical activity, lacunar infarcts,	All associations were made after adjustment for age, gender, education, lacunar infarcts and brain white matter volume. Additional adjustment for other covariates caused associations between only the highest number of total and periventricular white matter hyperintensities to remain significant; associations with deep white matter hyperintensities were no longer significant.
40. Stijntjes et al. (2016)	Older Adults (n = 237), age and gender characteristics not specified for just those with imaging and gait data	Walking speed (ms^−1^)	Timed walk (4 m and 25 m)	Total grey and white matter volumes, hippocampal volume, basal ganglia volume, cerebral microbleed presence, lacunar infarct presence	MRI: automated segmentation (SIENAX, FIRST) and visualization (MIPAV)	Slower walking speed associated with the presence of microbleeds and infarcts, and with smaller hippocampal volume (during 4 m walking only). No other associations between walking speed and total grey or white matter or basal ganglia volume was observed.	age, gender, whole brain volume, cognition through immediate and delayed recall	Associations were made with inclusion of age, gender and whole brain volume. Additional inclusion of cognition did not alter findings.
41. Tian et al. (2017b)	Older Adults (n = 59), 50.8%F, age 74.8 ± 7.8	Gait speed (ms^−1^)	Timed walks (6 m)	Amyloid beta burden through distribution volume ratio (DVR)	PET: [11C]PiB	No cross-sectional association between gait speed and amyloid beta burden was observed.	age, sex, BMI, cardiovascular risk, APOE ε4 status, California Verbal Learning Test score	Analyses included all covariates
42. Verlinden et al. (2016)	Older Adults (n = 2330), 55.1% F, age 65.9 ± 9.2	Gait velocity (cm/s), Stride length (cm), Single support phase (%), Stride length SD (cm)	GAITRite walkway (4.88 m)	Fractional Anisotropy (FA), Mean Diffusivity (MD), Axial Diffusivity (AD), Radial Diffusivity (RD)	DTI: Probabilistic tractography (ProbtrackX)	Slower gait velocity associated with increased MD in brainstem, projection, association, limbic and callosal tracts. Associations with FA occurred in all but brainstem tracts, with MD in all but limbic tracts, with RD in all tracts and with AD in association and projection tracts only. Shorter stride length associated with increased MD in brainstem, projection, association, limbic and callosal tracts. Reduced single support phase associated with MD in the anterior thalamic radiation (projection), inferior fronto-occipital fasiculus and superior longitudinal fasiculus (association). Increased stride length SD associated with increased MD in the superior longitudinal fasiculus (association) only	age, age-squared, sex, height, weight, education, interval between gait and MRI, MMSE, intracranial volume, lacunar infarcts, tract specific WM volume, log transformed WML volume, other gait domains, direction of encoding on scan	Associations were made with inclusion of covariates.
43. Wennberg et al. (2017a)	Older Adults (n = 611), 49.3% F, age 62.7 (age range 50-69)	Gait speed (ms^−1^), Stride length (cm), Stance time variability (%CV), Cadence (steps per minute), Double support time (s)	GAITRite (5.6 m)	Amyloid beta burden through standard uptake value ratios (SUVRs)	PET: [11C]PiB	Slower gait speed associated with higher amyloid beta burden in orbitofrontal and temporal regions. Stride length did not associate with amyloid beta burden in any region of interest. Increased stance time variability associated with higher amyloid beta burden in prefrontal, orbitofrontal, temporal, anterior and posterior cingulate and motor regions. Less cadence associated with higher amyloid beta burden in all regions of interest (orbitofrontal, prefrontal, parietal, temporal, anterior and posterior cingulate and motor regions). Increased double support time associated with increased amyloid beta burden in all regions except motor	age, sex, BMI, education, APOE e4 allele, Charlson comorbidity index, depression, AD-associated neurodegeneration (HVa, FDG PET SUVR, and cortical thickness)	Adjustment for AD-associated neurodegeneration caused gait speed to additionally associate with SUVR in prefrontal and anterior and posterior cingulate regions. Also, stride length associated with SUVR in temporal regions and stance time variability additionally associated with SUVR in parietal regions
44. Willey et al. (2013)	Older Adults (n = 701), 672% F, age 80.3 ± 5.6	Gait speed (ms^−1^)	Straight line walking (4 m)	WMH volume	MRI: Fluid-attenuated inversion recovery (FLAIR)	Slow gait speed associated with large total WMH volume in cross-section, as well as WMH volume in the frontal lobe.	age, ethnicity, gender, cardiovascular risk factors, MCI or dementia, silent brain infarcts	Associations were significant after adjusting for cardiovascular risk factors and silent brain infarcts, however adjustment for other covariates caused associations to be no longer significant
45. Wolfson et al. (2013)	Older Adults (n = 67), 61% F, age 81.7 ± 3.9	Gait velocity (ms^−1^)	SPPB (2.5 m)	WMH volume	MRI: Fluid-attenuated inversion recovery (FLAIR)	Slower gait velocity associated with total WMH volume at the baseline time point. No association was made at the second time point.	age, gender, BMI (baseline where appropriate)	Inclusion of all covariates in analyses had no effect
46. Yuan et al. (2015)	Older Adults (n = 30), 51.8% F, age 72.5 ± 5.2	Gait velocity (cm/s)	GAITRite (8 foot)	Functional connectivity	Resting-state fMRI: BOLD	Gait velocity associated with functional connectivity in sensorimotor, visual, vestibular, and left fronto-parietal cortical areas.	not reported	Unknown
47. Zimmerman et al. (2009)	Older Adults (n = 48), 47.9% F, age 81.2 ± 5.5	Stride length (cm), Stride length variability (SD)	GAITRite walkway (9 foot)	Hippocampal volume and hippocampal N-acetylaspartate: creatine ratio	MRI: egion of interest (ROI) Magnetic resonance spectroscopy: Gaussian broadening	Shorter stride length was associated with smaller hippocampal volume. There was no association between stride length or stride length variability and N-acetylaspartate: creatine ratio.	age, midsagittal area, gender, weight, gait velocity	Including gait velocity as a covariate caused the association to be no longer significant

**Fig. 4:**
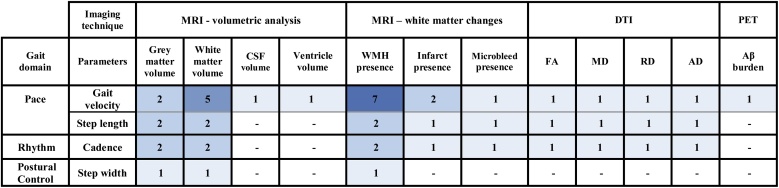
Heat map of the longitudinal studies assessing each imaging and gait parameter.

Eleven studies described longitudinal associations between gait and brain imaging parameters (Rosano et al., 2005b; Wolfson et al., 2005; Soumaré et al., 2009; Frederiksen et al., 2011; Ryberg et al., 2011; Moscufo et al., 2012; Callisaya et al., 2013; Willey et al., 2013; Wolfson et al., 2013; Tian et al., 2017b; van der Holst et al., 2018). Fig. 4 summarises the number of studies comparing each gait characteristic to each imaging parameter; more detailed descriptions of each longitudinal study are included in Table 3. Supplementary tables 3–7 demonstrate studies which found positive, negative or no longitudinal association between imaging parameters and gait.

**Table 3:** Descriptive information of all longitudinal studies.

Study	Participant characteristics	Gait variables measured longitudinally	Gait analysis tool	Imaging parameters measured	Imaging modality and analytical technique utilised	Time points at which gait and imaging parameters were assessed	Main Findings	Covariates	Effect of covariates on findings
1. Callisaya et al. (2013)	Older Adults (n = 225), 43.6% F, age 71.4 ± 6.8	Gait velocity (cm/s), Step length (cm), Cadence (steps per minute), Step width (cm)	GAITRite (4.6 m)	Total grey and white matter volume, WMH, hippocampal volume	MRI: automated segmentation, Fluid-attenuated inversion recovery (FLAIR)	Gait and MRI assessments were completed at both baseline and follow-up (mean duration between assessments was 30.6 ± 4.9 months.)	Greater decline in gait velocity over time associated with reduced grey matter, white matter and hippocampal volumes over time, and increased WMH volume over time. Greater decline in stride length over time associated with reductions in white matter and hippocampal volumes over time, and increased WMH volume over time. Greater decline in cadence over time associated with reduced grey and white matter volumes over time. Change in stride width over time did not associate with change in any imaging parameter over time	age, sex, BMI, total intracranial volume, time between assessments, change in grey matter/change in white matter/change in WMH as appropriate	Gait velocity only associated with grey matter volume reductions when baseline imaging parameters were included as covariates. Including age as a covariate modified the association between stride length decline and change in WMH volume over time; older adults had more strong associations. Cadence only associated with grey matter volume reductions when the number of infarcts at baseline was included as a covariate
2. Frederiksen et al. (2011)	Older Adults (n = 328), 53% F, age 73.8 ± 5.0	Gait velocity (ms^−1^)	Timed walk on unspecified walkway (8 m)	Corpus Callosum volume	MRI: automatic segmentation	Gait and MRI assessments were completed at both baseline and follow-up (additional gait and imaging assessments were conducted 4 years after baseline)	Change in gait velocity over time did not associate with change in total or regional corpus callosum volume over time	age, gender, Rotterdam progression scale score, MTA score, incident lacunes	Not reported
3. Moscufo et al. (2012).	Older Adults (n = 77), 60% F, age 84 ± 3.9	Walk speed (ms^−1^)	SPPB (2.5 m)	WMH volume	MRI: Fluid-attenuated inversion recovery (FLAIR)	Gait and MRI assessments were completed at both baseline and follow-up (mean duration between assessments was 1.9 ± 0.2 years.)	Change in gait velocity over time did not associate with change in total or regional WMH burden over time	age, gender, mini-mental state score (MMSE), and body mass index (BMI)	Inclusion of all covariates in analyses had no effect on associations
4. Rosano et al. (2005b)	Older Adults (n = 2450), 57% F, age 74.4 ± 4.7	Gait velocity (ms^−1^)	Timed walk (15 foot)	Ventricular enlargement, WMH volume, infarcts	MRI: comparison to an atlas of predefined visual standards	Gait and MRI assessments were completed baseline. At follow-up, only gait was re-assessed (mean duration between baseline and follow-up assessments was 4.0 years.)	A greater decline in gait velocity over time associated with greater ventricular enlargement, WMH volume and brain infarcts at baseline	age, sex, race, education, cardiovascular ris factors, cardiovascular disease	Association was made with inclusion of covariates
5. Ryberg et al. (2011)	Older Adults (n = 563), 55% F, age 74 ± 5	Gait velocity (ms^−1^)	Timed walk (8 m)	Corpus Callosum volume	MRI: automatic segmentation	Gait and MRI assessments were completed baseline. At follow-up, only gait was re-assessed (3 additional assessments of gait were conducted; 1, 2 and 3 years after baseline)	A greater decline in gait velocity over time associated with the interaction between corpus callosum volume at baseline and time; both total corpus callosum volume and regions 2 (Rostral body), 3 (Midbody) and 5 (Splenium)	age, gender, handedness, general atrophy, ARWMC load	Association was made with inclusion of covariates
6. Soumaré et al. (2009)	Older Adults (n = 1702), 60.6% F, age 72.4 ± 4.1	Walking speed (ms^−1^)	Chronometer connected to 2 photoelectric cells (6 m)	WMH volume	MRI: Fully automated software	Gait and MRI assessments were completed baseline. At follow-up, only gait was re-assessed (additional assessments of gait were conducted 8 years after baseline)	A greater decline in gait velocity over time associated with a high number of total and periventricular white matter hyperintensities at baseline.	age, gender, education, baseline walking speed, baseline white matter volume, baseline BMI, diabetes, physical activity, psychoactive drug use, lacunar infarcts at baseline	Inclusion of all covariates in analyses had no effect on associations
7. Tian et al. (2017b)	Older Adults (n = 59), 50.8% F, age 74.8 ± 7.8	Gait velocity (ms-1)	Course walks (6 m)	Amyloid beta burden through distribution volume ratio	PET: [11C]PiB	Gait and MRI assessments were completed baseline. At follow-up, only gait was re-assessed (mean duration between baseline and follow-up assessments was 4.7 years.)	A greater decline in gait velocity over time associated with higher amyloid beta burden at baseline, both in the overall cortex and regionally in the dorsolateral prefrontal cortex, putamen and lateral temporal lobe	age, sex, BMI, cardiovascular risk, APOE ε4 status, memory, depressive symptoms, peripheral arterial disease, processing speed, executive function, cerebrovascular disease ankle–arm index, DSST, TMT-B, and WMH/ICV, repeated measures of CVLT and CES-D.	Adjusting for TMT-B attenuated all associations except that with the putamen. Adjusting for DSST attenuated the association with dorsolateral prefrontal cortex
8. van der Holst et al. (2018)	Older Adults (with SVD, n = 275), 43.6% F, age 62.9 ± 8.2	Gait velocity (ms-1), Stride length (m), Cadence (steps per minute)	GAITRite (5.6 m)	Change in following characteristics: WMH volume, white matter volume, grey matter volume, infarcts, microbleeds, fractional anisotropy, mean diffusivity, axial diffusivity, radial diffusivity	MRI: Fluid-attenuated inversion recovery (FLAIR), SPM 12 DTI: Tract-Based Spatial Statistics (TBSS)	Gait and MRI assessments were completed at both baseline and follow-up (mean duration between assessments was 5.4 ± 0.2 years.)	A reduction in white matter volume was associated with a reduction in stride length. An increase in radial and mean diffusivity was associated with a decline in stride length; a decrease in mean fractional anisotropy related to increased decline of stride length. No other associations with stride length were made. No associations were made between gait velocity or cadence and any of the imaging parameters.	age, sex, follow-up duration, height, and baseline gait characteristic. Additionally, changes in cerebral small vessel disease characteristics for associations related to DTI parameters	Association was made with inclusion of covariates
9. Willey et al. (2013).	Older Adults (n = 701), 67.2% F, age 80.3 ± 5.6	Gait velocity (ms^−1^)	Timed walk (4 m)	WMH volume	MRI: Fluid-attenuated inversion recovery (FLAIR)	Gait and MRI assessments were completed baseline. At follow-up, only gait was re-assessed (mean duration between baseline and follow-up assessments was 4.7 ± 0.5 years.)	A decline to slow gait velocity over time associated with large total WMH volume at baseline, as well as WMH volume in the frontal lobe	age, ethnicity, gender, cardiovascular risk factors, MCI or dementia, silent brain infarcts, gait speed at enrollment, time between assessments	Associations were significant after adjusting for cardiovascular risk factors and silent brain infarcts, however adjustment for other covariates caused associations with WMH in the frontal lobe to be no longer significant
10. Wolfson et al. (2005)	Older Adults (n = 14), 36% F, age 81 ± 1.7 (normal mobility), age 84 ± 3.4 (impaired mobility)	Gait velocity (ms^−1^)	SPPB (2.5 m)	CSF volume, white matter volume	MRI: Fully automated software	Gait and MRI assessments were completed baseline. At follow-up, only gait was re-assessed (median duration between baseline and follow-up assessments was 20 months)	A change in gait velocity over time associated positively with total white matter volume and negatively with CSF volume at baseline	age and gait	Not reported
11. Wolfson et al. (2013)	Older Adults (n = 67), 61% F, age 81.7 ± 3.9	Gait velocity (ms^−1^)	SPPB (2.5 m)	WMH volume	MRI: Fluid-attenuated inversion recovery (FLAIR)	Gait and MRI assessments were completed at both baseline and follow-up (additional assessments of gait were conducted 4 years after baseline).	A change in gait velocity over time did not associate with either a change in WMH volume over time, or with baseline WMH volume	age, gender, BMI (baseline where appropriate)	Inclusion of all covariates in analyses had no effect

As anticipated, gait velocity was the gait characteristic most commonly assessed. Several characteristics of gait are yet to be associated with any brain imaging parameters, including gait speed variability, swing time asymmetry and stance time asymmetry. Only four gait characteristics, from three gait domains, have been assessed longitudinally; gait velocity, step length, cadence and step width. Gait velocity was the only characteristic considered longitudinally in all studies. Step width, from the postural control domain, was assessed in only one study, and was not associated with any imaging parameter longitudinally (Callisaya et al., 2013).

#### 3.3.1 MRI – volumetric analysis

##### 3.3.1.1 Cross-sectional associations from volumetric analysis

Measurements of brain volume were the most commonly derived brain imaging parameters; 21 studies evaluated associations between brain volumes and gait characteristics (Rosano et al., 2005a, b; Della Nave et al., 2007; Rosano et al., 2007a, 2008; Novak et al., 2009; Zimmerman et al., 2009; de Laat et al., 2011c; Sorond et al., 2011; de Laat et al., 2012; Dumurgier et al., 2012; Manor et al., 2012; Annweiler et al., 2014; Beauchet et al., 2014; Callisaya et al., 2014; Nadkarni et al., 2014; Rosso et al., 2014; Beauchet et al., 2015; Ezzati et al., 2015; Stijntjes et al., 2016; Beauchet et al., 2017).

###### 3.3.1.1.1 Grey matter volume

Grey matter (GM) volume was the most widely evaluated volume type, with 16 studies taking measures of GM atrophy (Rosso et al., 2014), cortical thickness as a proxy for GM volume (de Laat et al., 2012), or total (Della Nave et al., 2007; Manor et al., 2012; Callisaya et al., 2014), cortical (Ezzati et al., 2015; Stijntjes et al., 2016), or regional (Rosano et al., 2007a, 2008; Novak et al., 2009; Zimmerman et al., 2009; Dumurgier et al., 2012; Manor et al., 2012; Beauchet et al., 2014; Nadkarni et al., 2014; Beauchet et al., 2015; Ezzati et al., 2015; Stijntjes et al., 2016; Beauchet et al., 2017) GM volumes. Grey matter regions were identified either through manual region of interest identification or automated processes.

Fifteen of these studies evaluating GM volume assessed characteristics within the pace domain of gait. Eight of eleven studies assessing gait velocity found that slower gait was associated with reduced GM volume (Rosano et al., 2007a; Novak et al., 2009; de Laat et al., 2012; Dumurgier et al., 2012; Callisaya et al., 2014; Nadkarni et al., 2014; Ezzati et al., 2015; Stijntjes et al., 2016). This association was made across many brain regions. Slower gait was associated with reduced global GM volume (Callisaya et al., 2014; Ezzati et al., 2015), reduced frontal GM (Rosano et al., 2007a; Novak et al., 2009; Callisaya et al., 2014), reduced GM in the occipital cortex (Callisaya et al., 2014), reduced hippocampal volume (Ezzati et al., 2015; Stijntjes et al., 2016), and, in the only study to assess cortical thickness, with cortical thinning in all regions except for the inferior temporal gyrus (de Laat et al., 2012). Subcortically, slower gait was associated with cerebellar atrophy (Rosano et al., 2007a; Callisaya et al., 2014; Nadkarni et al., 2014), and reduced basal ganglia volume (Dumurgier et al., 2012; Callisaya et al., 2014). Reduced step length was also associated with reduced grey matter in all four studies assessing both parameters (Rosano et al., 2008; Zimmerman et al., 2009; de Laat et al., 2012; Callisaya et al., 2014). The association was again made with many brain regions, including global GM volume (Callisaya et al., 2014), hippocampal volume (Zimmerman et al., 2009), and prefrontal, parietal, supplementary motor, sensorimotor, occipital and limbic regional volumes (Rosano et al., 2008; de Laat et al., 2012). The relationship between step time variability and GM volume is less clear. Three of the four studies assessing this relationship used similar datasets; whilst greater step time variability was associated with increased hippocampal volume (Beauchet et al., 2015) and reduced GM volume of the right parietal lobe (Beauchet et al., 2014) in two papers, no association was made between step time variability and hippocampal or somatosensory volume in the third (Beauchet et al., 2017). Similarly, no association was made between stride duration variability and global GM volume or cerebellar, dorsolateral prefrontal cortex or basal ganglia volumes in one study (Manor et al., 2012). Swing time variability was not associated with hippocampal volume in one study (Beauchet et al., 2015); as no other studies have investigated swing time variability with grey matter volume, no firm conclusions can be drawn.

Five studies have investigated associations between GM volume and characteristics from the rhythm domain of gait. Two studies assessed double support time and had conflicting results; one found that longer double support time associated with a reduction of GM volume in areas including dorsolateral prefrontal cortex, right parietal lobules, right motor cortex and sensorimotor cortex (Rosano et al., 2008), the other found no association between double support time and global, cerebellar dorsolateral prefrontal cortex or basal ganglia GM volumes (Manor et al., 2012). Similarly, the three studies assessing step time and cadence – the inverse of step time – had conflicting results. Whilst no association was made between step time and hippocampal volume (Beauchet et al., 2015), or between cadence and total GM volume (Callisaya et al., 2014), one study found that decreased cadence was associated with cortical thinning in left cingulate and visual regions, the left fusiform gyrus and the primary and premotor cortices (de Laat et al., 2012). Only one study assessed swing time; it was not associated with hippocampal volume (Beauchet et al., 2015).

Within the variability domain, one study investigated the association between each of step length variability and step width variability; these were not associated with total grey matter atrophy (Rosso et al., 2014) or hippocampal volume (Beauchet et al., 2015). No gait characteristics within the asymmetry domain have yet been assessed with GM volume. Three studies assessed GM volume with step width, the only characteristic assessed from the postural control domain. Two of three studies found that wider steps were associated with reduced GM volume in the inferior parietal lobe (Rosano et al., 2008; de Laat et al., 2012). Several other brain regions were related to step width in only one of these two studies, including the orbitofrontal and ventrolateral prefrontal cortices, temporal gyrus, left fusiform gyrus and the dorsal anterior cingulate cortex (de Laat et al., 2012), as well as the pallidum and right dorsolateral prefrontal cortex (Rosano et al., 2008).

###### 3.3.1.1.2 White matter volume

Six studies assessed total, cortical and regional white matter (WM) volumes (Della Nave et al., 2007; Sorond et al., 2011; Beauchet et al., 2014; Callisaya et al., 2014; Ezzati et al., 2015; Stijntjes et al., 2016). All studies assessed characteristics from the pace domain of gait; one additionally assessed cadence from the rhythm domain. Four of five studies found no association between WM volume and gait velocity (Della Nave et al., 2007; Callisaya et al., 2014; Ezzati et al., 2015; Stijntjes et al., 2016). Similarly, no association was identified between WM volume and step length (Callisaya et al., 2014) or step time variability (Beauchet et al., 2014) from the pace domain, or with cadence from the rhythm domain (Callisaya et al., 2014). No other gait characteristics were assessed with WM volume.

###### 3.3.1.1.3 Total brain volume

Total brain volume was assessed in three studies, either as an absolute value (Della Nave et al., 2007; de Laat et al., 2011c) or through assessment of brain parenchymal fraction (Sorond et al., 2011). All assessed gait velocity; two of three studies found no association between gait velocity and total brain volume (Della Nave et al., 2007; Sorond et al., 2011). De Laat et al., however, found associations between total brain volume and gait characteristics from all domains except asymmetry (de Laat et al., 2011c) with increased total brain volume being associated with increased gait velocity, step length and cadence, and with reduced step time variability, double support time, step length variability and step width.

###### 3.3.1.1.4 Ventricular volume

Four studies assessed ventricular volume (Rosano et al., 2005a, b; Annweiler et al., 2014; Ezzati et al., 2015). Again, all studies assessed gait characteristics within the pace domain of gait, and one additionally assessed double support time from the rhythm domain. Gait velocity was the only gait characteristic assessed in multiple studies; two of these three studies found no association between gait velocity and ventricular volume (Rosano et al., 2005a; Ezzati et al., 2015). For the other gait characteristics assessed, one study found that increased step time variability was associated with increased ventricular volume (Annweiler et al., 2014), whereas another study found no association between ventricular volume and either step length or double support time (Rosano et al., 2005a). No characteristics from the variability, asymmetry or postural control domains were assessed with ventricular volume.

##### 3.3.1.2 Longitudinal associations from volumetric analysis

###### 3.3.1.2.1 Grey matter volume

In contrast to the cross-sectional findings, only two studies assessed changes in GM volume with changes in gait (Callisaya et al., 2013; van der Holst et al., 2018), and their results conflicted. Van der Holst et al. found no association between GM volume change and a change in either gait velocity, step length or cadence (van der Holst et al., 2018), whereas Callisaya et al. identified that a decrease in total GM volume over time was associated with a decrease in gait velocity and a decrease with cadence over time, and hippocampal volume reduction was associated with slowing of gait velocity and shortening of step length over time (Callisaya et al., 2013).

###### 3.3.1.2.2 White matter volume

Five studies assessed WM volume longitudinally with gait (Wolfson et al., 2005; Frederiksen et al., 2011; Ryberg et al., 2011; Callisaya et al., 2013; van der Holst et al., 2018). Both studies that used baseline WM volumes to predict gait changes identified that smaller baseline WM volume were predictive of greater decline in gait velocity over time (Wolfson et al., 2005; Ryberg et al., 2011). However, of the three studies assessing a change in WM volume with a change in gait velocity (Frederiksen et al., 2011; Callisaya et al., 2013; van der Holst et al., 2018), only one found that greater reductions in WM volume over time related to greater decline in gait velocity (Callisaya et al., 2013). A reduction in step length over time was associated with a reduction in WM volume over time in both studies assessing it (Callisaya et al., 2013; van der Holst et al., 2018). Change in cadence, from the rhythm domain of gait, was assessed with WM volume change in two studies (Callisaya et al., 2013; van der Holst et al., 2018), yet results were conflicting. One study found that a reduction in total WM volume over time was associated with a reduction in cadence over time (Callisaya et al., 2013), whereas another study found no association between the parameters (van der Holst et al., 2018).

###### 3.3.1.2.3 CSF and ventricular volume

Two studies used baseline CSF volume (Wolfson et al., 2005) and ventricular volume (Rosano et al., 2005b) to predict gait changes; larger volumes of each of these at baseline were predictive of a greater decline in gait velocity over time.

#### 3.3.2 MRI – white matter changes associated with age

##### 3.3.2.1 Cross-sectional associations from analysis of white matter change

20 studies investigated cross-sectional associations between gait and white matter changes which are common in ageing, namely a combination of white matter hyperintensity (WMH) presence, which includes age-related white matter changes (Baezner et al., 2008) leukoaraiosis (Della Nave et al., 2007) and measures of WMH presence and severity (Rosano et al., 2005a, b; Rosano et al., 2007b; Baezner et al., 2008; Nadkarni et al., 2009; Novak et al., 2009; Soumaré et al., 2009; Murray et al., 2010; de Laat et al., 2011a; Sorond et al., 2011; Moscufo et al., 2012; Willey et al., 2013; Wolfson et al., 2013; Bolandzadeh et al., 2014; Rosso et al., 2014; Rosario et al., 2016; Stijntjes et al., 2016), a presence of infarcts (Rosano et al., 2005a, b; Rosano et al., 2007b; Choi et al., 2012; Stijntjes et al., 2016) and a presence of microbleeds (de Laat et al., 2011b; Choi et al., 2012; Stijntjes et al., 2016).

###### 3.3.2.1.1 WMH presence

WMH presence was commonly assessed, with 18 studies evaluating it with gait (Rosano et al., 2005a, b; Della Nave et al., 2007; Rosano et al., 2007b; Baezner et al., 2008; Nadkarni et al., 2009; Novak et al., 2009; Soumaré et al., 2009; Murray et al., 2010; de Laat et al., 2011a; Sorond et al., 2011; Moscufo et al., 2012; Willey et al., 2013; Wolfson et al., 2013; Bolandzadeh et al., 2014; Rosso et al., 2014; Rosario et al., 2016; Stijntjes et al., 2016).

Seventeen studies assessed WMH presence with gait characteristics from the pace domain; only one study did not assess gait velocity (Della Nave et al., 2007). Findings within the domain were not consistent with each other; 13 of 16 studies found that slower gait velocity was associated with increased WMH presence (Rosano et al., 2005a, b; Baezner et al., 2008; Nadkarni et al., 2009; Soumaré et al., 2009; Murray et al., 2010; de Laat et al., 2011a; Sorond et al., 2011; Moscufo et al., 2012; Willey et al., 2013; Wolfson et al., 2013; Bolandzadeh et al., 2014; Rosario et al., 2016), whereas two of three studies found no association between step length and WMH presence (Rosano et al., 2005a; Nadkarni et al., 2009). Regionally, reduced gait velocity was linked to more WMHs within the corpus callosum, both in anterior (Bolandzadeh et al., 2014; Rosario et al., 2016) and posterior (Moscufo et al., 2012) regions, frontal brain regions (Nadkarni et al., 2009; Murray et al., 2010; Willey et al., 2013), periventricular regions (Soumaré et al., 2009; Murray et al., 2010) and within the basal ganglia (Rosano et al., 2005a; Nadkarni et al., 2009). Other regions of WMH presence were associated with gait velocity, including the right centrum semiovale (de Laat et al., 2011a), the anterior corona radiata, superior longitudinal and fronto-occipital fasciculus (Rosario et al., 2016) the right anterior thalamic radiation (Bolandzadeh et al., 2014) and temporal, parietal and occipital regions (Murray et al., 2010). One study assessed stance time variability, and found that increased stance time variability was associated with increased presence of WMHs (Rosano et al., 2007b).

Three studies have assessed WMH presence with characteristics from the rhythm domain of gait. Again, findings within the domain were inconsistent with each other. Whilst two studies found no association between WMH presence and cadence (Nadkarni et al., 2009; de Laat et al., 2011a), the other found that increased global WMH presence, and WMHs within the brainstem, were associated with increased double support time (Rosano et al., 2005a).

Two studies have assessed WMH presence with gait characteristics from the variability domain. One study found that increased WMHs were associated with an increase in step length variability, but not with step width variability (Rosano et al., 2007b). The other study found no association between WMH presence and step length variability (Rosso et al., 2014). No gait characteristics within the asymmetry domain have yet been assessed with WMH presence. Two studies assessed WMHs with step width, from the postural control domain of gait, and found conflicting results. Whilst one study found that increased WMH presence was associated with wider steps in several brain regions (de Laat et al., 2011a), the other found that increased WMH presence was associated with narrower steps within the basal ganglia (Nadkarni et al., 2009).

###### 3.3.2.1.2 Infarct presence

Five studies assessed infarct presence with gait (Rosano et al., 2005a, b; Rosano et al., 2007b; Choi et al., 2012; Stijntjes et al., 2016). Each of these studies assessed gait characteristics from the pace domain; all four studies assessing gait velocity found that slower gait related to a higher infarct presence (Rosano et al., 2005a, b; Choi et al., 2012; Stijntjes et al., 2016), one of which reported that infarcts in the basal ganglia were associated with gait velocity (Rosano et al., 2005a). Both studies assessing step length associated shorter steps with an increased infarct presence (Rosano et al., 2005a; Choi et al., 2012). One study assessed stance time variability, which was not associated with either the number of total or basal ganglia infarcts (Rosano et al., 2007b).

Associations between an infarct presence and characteristics from the other gait domains are sparse and inconclusive. Two studies assessed infarct presence with double support time, yet only one found that increased infarct presence associated with increased double support time (Choi et al., 2012). This study also found that a higher presence of infarcts was associated with increased step width, but not cadence. The only study to investigate gait characteristics from the variability domain found that an increased infarct presence, both globally and within the basal ganglia, was associated with step length variability, but not step width variability (Rosano et al., 2007b).

###### 3.3.2.1.3 Microbleed presence

Three studies assessed a presence of microbleeds with gait (de Laat et al., 2011b; Choi et al., 2012; Stijntjes et al., 2016). All three studies assessed gait velocity, yet only two found that an increased microbleed presence was associated with reduced gait velocity (de Laat et al., 2011b; Stijntjes et al., 2016). De Laat et al. specified that this association was made not only with a global presence of microbleeds, but with microbleeds within the temporal lobe (de Laat et al., 2011b). Two of the three studies assessed additional gait characteristics (de Laat et al., 2011b; Choi et al., 2012). Both found that an increased presence of microbleeds was associated with increased double support time, but their results conflicted whilst assessing associations with step length, cadence and step width.

##### 3.3.2.2 Longitudinal associations from analysis of white matter change

###### 3.3.2.2.1 WMH presence

WMH presence was the most investigated imaging parameter longitudinally; seven studies longitudinally assessed WMHs with gait (Rosano et al., 2005b; Soumaré et al., 2009; Moscufo et al., 2012; Callisaya et al., 2013; Willey et al., 2013; Wolfson et al., 2013; van der Holst et al., 2018). All seven studies assessed WMHs with gait velocity. In keeping with findings from cross-sectional evaluations, three of four studies that assessed baseline WMH presence with change in gait velocity found that increased WMH presence at baseline was associated with a greater decline in gait velocity over time (Rosano et al., 2005b; Soumaré et al., 2009; Willey et al., 2013). However, only one of three studies assessing whether a change in the number of WMHs over time was associated with a longitudinal change in gait velocity found that a higher accumulation of WMHs over time was associated with a greater decline in gait velocity (Wolfson et al., 2013). Two studies assessed a change in WMH presence with a change in step length; one found that increased WMH presence over time was associated with slowing of gait velocity over time (Callisaya et al., 2013), whereas the other found no association (van der Holst et al., 2018). Neither of these studies found an association between a change in the number of WMHs and a change in cadence.

###### 3.3.2.2.2 Infarct and microbleed presence

Two studies assessed the longitudinal associations between gait and infarct and/or microbleed presence. One study found that a higher presence of infarcts at baseline was associated with a greater decline in gait velocity (Rosano et al., 2005b), whereas the other found no association between a change in either infarct or microbleed presence and a change in gait velocity, step length or cadence (van der Holst et al., 2018).

#### 3.3.3 DTI

##### 3.3.3.1 Cross-sectional associations with DTI parameters

Eight studies have utilised DTI imaging parameters in cross-sectional analyses (Della Nave et al., 2007; de Laat et al., 2011a, c; Bruijn et al., 2014; Rosso et al., 2014; Fling et al., 2016; Verlinden et al., 2016; Fling et al., 2018). A variety of methods were used to determine DTI parameters, including Tract-Based Spatial Statistics (TBSS), Interhemispheric callosal tractography, Probabilistic tractography through ProbtrackX, SPM5 or in-house software.

###### 3.3.3.1.1 Fractional anisotropy

All studies utilizing DTI determined fractional anisotropy (FA); high values of FA indicated a good integrity of the tracts assessed. Five investigated gait characteristics from the pace domain; three of these five found that slower gait velocity was associated with reduced FA (de Laat et al., 2011a, c; Verlinden et al., 2016) in most white matter tracts, except for the brainstem, in one study (Verlinden et al., 2016) and expect for periventricular regions in another (de Laat et al., 2011c). Both studies assessing step length found that shorter steps were associated with FA (de Laat et al., 2011a, c), and the one study that assessed step time variability found that increased variability was associated with reduced FA (de Laat et al., 2011c). Two studies assessed gait characteristics from the rhythm domain (de Laat et al., 2011a, c); both found that less cadence was associated with reduced FA. Only one of these studies assessed double support time, and found that it was not associated with FA (de Laat et al., 2011c). Two studies assessed characteristics from the variability domain. Whilst one found that increased step length variability was associated with reduced FA within grey matter (Rosso et al., 2014), the other found no association between FA in white matter and either step length variability or step width variability (de Laat et al., 2011c). The recent study from Fling et al. was the only one to assess step time asymmetry, from the asymmetry domain, or step length asymmetry from the postural control domain (Fling et al., 2018). Neither characteristic was associated with FA. Four studies assessed step width with FA, and their results conflicted. Two studies found that wider steps were associated with reduced FA (de Laat et al., 2011a, c), one found that narrower steps associated with reduced FA (Bruijn et al., 2014) and the other study found no association between the parameters (Fling et al., 2016).

###### 3.3.3.1.2 Mean, radial and axial diffusivity

Four studies additionally used measures of mean diffusivity (MD) within white matter tract, where high MD is a sign of poor white matter integrity (Della Nave et al., 2007; de Laat et al., 2011a, c; Verlinden et al., 2016). Three studies found that increased MD was associated with both slower gait velocity and shorter step length from the pace domain, and with increased step length variability from the variability domain (de Laat et al., 2011a, c; Verlinden et al., 2016). Associations with MD were made in more regions than those with FA assessment; associations were additionally made in subcortical frontal and parietal regions in one study (de Laat et al., 2011c), and within the brainstem in another study (Verlinden et al., 2016). Two studies assessed additional gait characteristics (de Laat et al., 2011a, c); both found that increased MD was associated with reduced cadence from the rhythm domain and increased step width from the postural control domain. Additionally, one of these studies identified that increased MD was associated with increased step time variability (pace) and increased double support time (rhythm), but was not associated with step width variability (variability) (de Laat et al., 2011c). Another study found that increased MD was associated with short stance time (rhythm) (Verlinden et al., 2016). Two of the studies assessing MD additionally reported associations relating to radial diffusivity (RD) and axial diffusivity (AD) (de Laat et al., 2011a; Verlinden et al., 2016), which related to gait in the same way as MD in both studies.

##### 3.3.3.2 Longitudinal associations with DTI parameters

One study utilised DTI (van der Holst et al., 2018) to determine longitudinal neural imaging correlates of gait. Van der Holst et al. found that changes in DTI parameters over time were not associated with decline in gait velocity or cadence, but that a decrease in FA over time was associated with a reduction in step length over time (van der Holst et al., 2018). Similarly, reduced step length over time was associated with increased MD and RD over time, but not increased AD.

#### 3.3.4 PET

##### 3.3.4.1 Cross-sectional associations with PET imaging

Four studies assessed amyloid beta, Aβ, burden through PET imaging (del Campo et al. (2016); Nadkarni et al., 2017; Tian et al., 2017b; Wennberg et al., 2017a). Three of these utilised [^11^C] Pittsburgh Compound B (PiB) PET scans (Nadkarni et al., 2017; Tian et al., 2017b; Wennberg et al., 2017a), whereas del Campo et al. used [18 F] Florbetapir PET scans (del Campo et al., 2016). Three of the studies found that an increased burden of Aβ was associated with reduced gait velocity (del Campo et al., 2016; Nadkarni et al., 2017; Wennberg et al., 2017a). Specifically, Aβ burden within basal ganglia regions, the precuneus, the temporal cortex and the anterior cingulate was negatively associated with gait velocity in more than one of these studies. No association was identified between gait velocity and Aβ burden within the hippocampus, pons, thalamus or parietal lobe. Additionally, Wennberg et al. (Wennberg et al., 2017a) found that increased Aβ burden was associated with increased stance time variability, reduced cadence and increased double support time, in prefrontal, temporal and cingulate regions; no associations were made between step length and Aβ burden.

Three studies used FDG PET imaging to determine cerebral glucose uptake (Shimada et al., 2013; Sakurai et al., 2014, 2017). All three of these studies recruited only female participants from similar databases of elderly volunteers, limiting an overall interpretation of the associations between cerebral glucose uptake and gait in the general population. Both of the studies authored by Sakurai et al. (Sakurai et al., 2014; Sakurai et al., 2017) found that lower uptake of glucose was associated with reduced gait speed and reduced cadence, but not step length. Associations were made in the posterior cingulate and parietal cortex in both studies; other regions were investigated, but either no association was made or the associations was not consistent across both studies. These associations were made with gait characteristics assessed during fast walking only; no associations were made between glucose uptake and gait characteristics measured at a comfortable walking pace. In the study from Shimada et al. (Shimada et al., 2013), participants were split in to two groups, those with low step length variability (LSV) and those with high step length variability (HSV). The LSV group had relatively increased glucose uptake in the primary sensorimotor area in comparison to the HSV group, and the HSV group had comparatively decreased uptake in the middle and superior temporal gyrus and hippocampus in relation to the LSV group; note that the direction of association is consistent in both sets of analyses. In this study, step length variability was assessed during walking at a fixed pace of 2 km/h, a relatively slow walking speed (Bohannon and Williams Andrews, 2011).

##### 3.3.4.2 Longitudinal associations with PET imaging

One study utilised PET imaging to determine longitudinal neural gait correlates. Gait velocity change was the only gait characteristic assessed alongside baseline PET imaging; Tian et al. found that higher baseline Aβ burden was associated with a greater slowing of gait velocity over time (Tian et al., 2017b).

#### 3.3.5 fNIRS, MRS and fMRI

Only one study using each of fNIRS (Holtzer et al., 2015), fMRI (Yuan et al., 2015) and MRS (Zimmerman et al., 2009) imaging techniques in relation to single-task gait characteristics were identified in this review. All studies were performed in cross-section, and assessed gait characteristics from the pace domain. These studies identified that gait velocity was associated with functional connectivity in sensorimotor, visual, vestibular, and left fronto-parietal cortical areas (Yuan et al., 2015), but not with activation strength of the prefrontal cortex (Holtzer et al., 2015), and stride length was not associated with either prefrontal cortex activation or N-acetylaspartate:creatine ratio within the hippocampus (Zimmerman et al., 2009). However, the sparsity of studies focussed on these approaches and gait means that no firm conclusions can be drawn about associations between gait and parameters derived from these functional imaging techniques.

### 3.4 The effect of cognition on the relationship between gait and imaging correlates

The impact of cognition on the relationship between gait and imaging correlates was considered, through assessment of studies which assessed cognition and included cognitive test scores as covariates. Twelve studies within this review included a measure of cognition as a potential confounding factor (Rosano et al., 2008; Dumurgier et al., 2012; Annweiler et al., 2014; Bolandzadeh et al., 2014; Nadkarni et al., 2014; Rosso et al., 2014; Sakurai et al., 2014; Ezzati et al., 2015; Stijntjes et al., 2016; Beauchet et al., 2017; Nadkarni et al., 2017; Tian et al., 2017b). These cognitive measures included global and primarily executive outcomes: the mini mental state exam (MMSE), the modified mini mental state exam (3MS), trail making tasks A and B (TMT-A, TMT-B), the digit symbol substitution test (DSST), the frontal assessment battery (FAB) and the abbreviated Stroop test, although one study specifically included a free recall score task (Stijntjes et al., 2016).

Gait velocity was frequently assessed as a measure in these studies. Adjusting for cognition caused gait velocity to no longer be associated with frontal and parietal lobe GM volume (Dumurgier et al., 2012), hippocampal volume (Ezzati et al., 2015), cerebellar volume (Nadkarni et al., 2014), amyloid beta burden (Nadkarni et al., 2017), WMHs in the anterior thalamic radiation and anterior corpus callosum (Bolandzadeh et al., 2014) and almost all regional associations with baseline amyloid beta burden (Tian et al., 2017b). In contrast, gait velocity remained associated with some imaging parameters after the inclusion of cognition as a covariate, in particular, with basal ganglia and caudate volumes (Dumurgier et al., 2012) as well as global microbleed and infarct presence (Stijntjes et al., 2016).

The effect of cognition on the neural correlates of other gait characteristics were also considered. Associations between regional grey matter volume and step length, stance time and step width (Rosano et al., 2008) were largely unaffected by the inclusion of cognition in statistical models, as were associations made between mean diffusivity in grey matter and step length variability (Rosso et al., 2014) and the association between step time variability and ventricular volume (Annweiler et al., 2014).

## 4 Discussion

To our knowledge, this is the first structured review to comprehensively map many discrete gait characteristics, defined by a validated objective gait model, to their structural and functional imaging neural correlates. We identified many associations between gait characteristics and the brain, however, our findings demonstrate a limited understanding of the overall neural control of gait. This is as a result of studies mostly reporting associations with gait velocity, and the majority of studies focussing on structural, rather than functional, neuroimaging parameters. Additionally, network-based approaches were scarcely utilised within the neuroimaging methodologies, despite the importance of networks becoming increasingly evident (O’Sullivan et al., 2001). There is emerging evidence linking other gait characteristics to a wider array of neuroimaging parameters, and of the effect of the ageing process on associations through longitudinal observations, although this remains limited.

### 4.1 Global neuroimaging correlates of discrete gait characteristics

The overarching findings from the studies included in this review is that a ‘healthy brain’ is associated with better gait. Larger volumes of healthy grey matter and WM integrity, determined through WMH presence in addition to FA and MD, were consistently associated with quality of gait performance, as demonstrated by gait characteristics related to pace, rhythm, variability and postural control. Increased step width, from the postural control domain, was associated with both greater and less white matter integrity in different studies, consistent with the idea that optimal step width is neither too wide nor too narrow. Although wide steps provide greater stability (Gabell and Nayak, 1984), they could indicate compensation for poor balance control. Non-linear optimisation of step width may also begin to explain why step width variability has not yet been associated with any imaging parameter.

The global presence of common white matter changes in ageing was associated with worse gait. Several of the studies in this review found that associations between gait and either brain volumes or DTI parameters were no longer significant once the presence of white matter changes such as WMH, infarct or microbleed presence were included as covariates, either in isolation or with a combination of other confounding factors. Some studies specified the presence of small vessel disease (through WMH or infarct presence) within their inclusion criteria (de Laat et al., 2011a, b, c; de Laat et al., 2012; van der Holst et al., 2018), and none of the studies included assessments related to ageing factors such as frailty or muscle mass. Overall, this causes great difficulty in discerning whether global neural gait correlates are due only to the presence of white matter artefacts, or if in fact these artefacts are an unrelated sign of ageing, and we are simply identifying associations between ageing and gait. Clearer assessment of the direct effects of age-related white matter changes on associations between gait and the brain should be completed in future to resolve this issue. The global imaging correlates of gait presented here suggest that gait may not controlled by discrete regions, instead many different regions may be working harmoniously for effective gait.

### 4.2 Regional neural correlates of gait

**Fig. 5:**
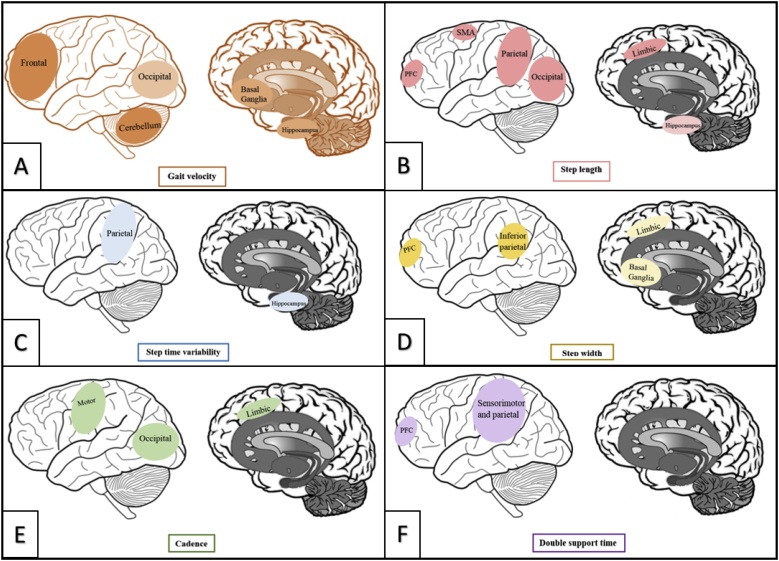
Map of the regional associations between GM volume and gait characteristics; gait velocity (A), step length (B), step time variability (C), step width (D), cadence (E) and double support time (F). Areas which are darker in colour indicate regions that were associated with the characteristic in multiple studies. Panel A shows the entire brain in an orange colour, to indicate that the volume of most brain regions have been associated with gait velocity.

In addition to global associations between gait and the brain, we aimed to identify regional neural structures and/or functions which related to specific gait characteristics. Studies which have attempted regional analyses were too disparate in approach to allow any firm conclusions to be drawn. A number of studies have focussed only on one region and most studies have each investigated different regions. Very few studies within this review assessed regions connected together through a network-based approach, either through the assessment of functional networks (Yuan et al., 2015) or by assessing the specific structural tracts which connect different brain regions (Verlinden et al., 2016). We have identified several trends of gait domains relating to specific brain regions; it is important to highlight, however, that greater efforts should now be made to assess these regions together as one or several networks which may be responsible for gait control. Within the pace domain, the most consistent trends were towards an association of gait velocity with GM volume in frontal, basal ganglia, hippocampal and cerebellar regions, and with WMHs within frontal regions, the basal ganglia and the corpus callosum, although most brain regions have been associated with gait velocity in at least one study. Step length was consistently associated with several GM volumes, however, not all the regions associated with gait velocity also related to step length. This suggests specificity in findings; gait velocity, as the global measure of gait, may be associated with global brain features, whereas step length may be related more so to cortical, rather than subcortical brain regions. For gait characteristics from the other domains, few studies specified the regions in which associations were made, and most regions were identified in only one study. Fig. 5 shows maps of the regional associations between GM volume and all gait characteristics in which GM regions were specified; gait velocity, step length, step time variability, step width, cadence and double support time. The entirety of the gait velocity maps are in colour, to indicate that most brain regions have been associated with gait velocity in at least one study; regions in which several associations have been made have additionally been highlighted.

It is clear from Fig. 5 that some brain regional volumes have been associated with several gait characteristics. This may be due to the volumes assessed being too crude, which would not allow for the specificity of associations between smaller, better defined, regions and gait to be apparent. Alternatively, it may be that brain regions are responsible for several of the gait characteristics, which could be grouped in to their own region-dependent domains in future. Before this can be clarified, however, further regional analyses assessing many of the gait characteristics through the same methodologies are required.

In studies assessing infarct and microbleed presence, the topographic location of these brain insults were rarely reported, although from the few studies that did detail regional associations it appears that insults within the basal ganglia have a negative impact on gait. Similarly, amyloid beta burden within the basal ganglia was associated with gait velocity. It is not yet clear whether these disturbances to the basal ganglia impact on all aspects of gait; it may be hypothesised that the basal ganglia is responsible for less complex gait characteristics, such as step length, as it is a lower level structure, although there is currently not enough literature to determine this with any certainty.

Two other reviews have recently been published which sought to determine neural gait correlates. Tian et al. focussed on associations made with variability characteristics of gait, and identified a particular association with temporal variability measures and the right hemisphere (Tian et al., 2017a). Here, we identified that the right hemisphere may also have a greater responsibility for double support time (Rosano et al., 2008), whereas cadence may be more dependent on the left hemisphere (de Laat et al., 2012); this asymmetry gives further evidence for specificity in the brain regions responsible for gait, and should be considered further. Wennberg et al. found that frontal and parietal regions of grey matter were most commonly associated with gait, as measured through more complex tests such as the timed up-and-go in addition to some of the gait characteristics identified here (Wennberg et al., 2017b)., Here we additionally found that the involvement of subcortical areas such as the basal ganglia and limbic system, as well as the hippocampus, were related to several gait characteristics. Several of the gait characteristics appeared to rely on involvement from both types of brain area, strengthening the argument that brain areas associated with both motor tasks and cognition are heavily involved in coordinating gait.

This review has demonstrated that, although many brain regions have been assessed with gait, there is a lack of cohesion between studies about the brain areas of most importance in gait. The non-specificity of findings between gait and structural volumes suggests that the impact of gait impairment on neural networks, connected either structurally or functionally, should be considered in future.

### 4.3 Longitudinal neural correlates of gait

A reduction in gait characteristics from the pace domain over time was not only associated with changes in WM volume over time, but also with baseline measures of WM volume. However, WM volume was not associated with gait during cross-sectional analyses. This highlights the importance of longitudinal study types when assessing associations between gait and the ageing brain, yet relatively few studies assessed the longitudinal neural correlates of gait. In general, we identified more associations between gait changes over time and imaging parameters when imaging was used as a *predictive* measure of gait decline, rather than where changes in imaging parameters were related to gait changes. This may indicate that predictive models utilising only one set of brain imaging data should be considered more regularly in future, particularly given that gait assessment is relatively inexpensive and is more accessible to older adults than imaging. Clinically, improved understanding of the brain changes associated with a poorer gait performance would allow us to utilise gait assessment more readily as a predictive measure for future neurodegeneration.

### 4.4 Methodological critiques

Most studies included in this review utilised structural MRI and/or DTI protocols, perhaps due to the major issue of cost surrounding functional imaging techniques such as PET and fNIRS and the intensity of task-based fMRI studies. Most studies that have utilised fMRI to assess associations between the brain and gait have done so through finding the neural correlates of imagined walking and leg movements whilst in an fMRI scanner. These were not considered in this review, as these protocols do not directly explore discrete gait characteristics. The use of virtual reality and foot pedals within functional protocols may provide further insight in to the functional networks utilised during walking, particularly as recent work has started to produce surrogate measures of gait characteristics such as step time variability (Gilat et al., 2017). The recent development of new radiotracer elements and enhanced PET scanning techniques may contribute to our understanding of neural activity associated with gait. For example, in healthy adults aged 21–85 years, gait velocity, cadence, and stance time have been slowed in those with lower striatal dopamine activity (Cham et al., 2008), and in PD, acetylcholinesterase ([11C]PMP) PET has been used to show that cholinergic deficit is associated with slow gait (Müller et al., 2015). Additionally, nerve stimulation can be used to assess cholinergic activity through assessment of short-latency afferent inhibition (Rochester et al., 2012); consideration of nerve or brain stimulation was beyond the scope of this review. To the best of our knowledge, no studies have utilised functional imaging of neurotransmitter activity in a group of healthy older adults to assess neural correlates of gait. Four of the seven studies which used PET imaging in this review assessed amyloid burden which, in effect, is a marker of structural rather than functional pathology and does not enhance our understanding of the functional neurochemical correlates underlying gait impairments. Although the assessment of CSF biomarkers through lumbar puncture was not covered here, studies of this nature can provide us with additional information about the effects of an accumulation of pathologies such as amyloid and tau on motor performance (Rochester et al., 2017).

Only one study within this review completed brain imaging in real time during the assessment of discrete gait characteristics. This is due to most fNIRS and EEG studies comparing differences in brain states between tasks, such as between single and dual task gait, rather than assessing single task gait characteristics (Hamacher et al., 2015; Vitorio et al., 2017). Electrophysiological responses, as measured through EEG, are consistent with haemodynamic responses assessed through both fNIRS and fMRI (Anwar et al., 2016), demonstrating their reliability as promising technologies for future research in to the functional neural correlates of gait. Additionally, functional imaging both during real-time gait assessment and during the resting state can be used to assess neural networks; it is becoming increasingly evident that network analyses, particularly those of a dynamic rather than static nature, will be key to furthering our understanding of the system-wide neural substrates which underpin dynamic gait control. There is evidence of an interaction between gait velocity and inter-network connectivity between the default mode network and supplementary motor as assessed through fMRI in older adults with mild cognitive impairment (Crockett et al., 2017), yet no study has assessed this in a healthy ageing population; these areas could provide a starting point for the neural networks to assess in association with gait. Decoupling between imaging and gait measurements introduces the potential for increased variance and noise, undermining potential correlation. Although structural imaging approaches are likely to be robust to this, functional imaging may be more sensitive due to its reliance on brain state and performance. To avoid this issue, further efforts should be made to develop strong protocols assessing real-time brain function with single task gait characteristics.

Several of the gait protocols within this review included use of a stopwatch during corridor walking. Although measures of gait velocity can be calculated through this technique, and its components in some instances, more subtle gait characteristics cannot be assessed. This limits our understanding of the neural control of variability and asymmetry; measures within the variability domain particularly have a high prevalence within gait research as they can be used as markers of fall risk and cognitive decline (Montero-Odasso et al., 2012; Tian et al., 2017a), therefore it is crucial that we develop a greater understanding of their underlying mechanisms. Additionally, non-linear approaches to analysis, such as the fractal analysis of stride-to-stride fluctuations in walking, are of increasing interest and prevalence within human gait research as they take in to consideration the structure and complexity of these large data sets (Hu et al., 2009; Li et al., 2018). Studies within this review varied not only by the gait measurement tools, but by the number, speed and type (continuous or intermittent) of walks performed, limiting our interpretation of findings. If a standard robust single-task gait protocol were developed and used, findings would be more comparable between studies, and standardised reporting of characteristics other than gait velocity would be carried out more frequently.

### 4.5 Cognition as a covariate

Cognition had a mediating effect on some of the associations made between gait velocity and volumes of the frontal lobe and hippocampus. Executive function (the function of which is subsumed by frontal regions) and memory (controlled by the hippocampus as well as portions of the prefrontal cortex) have both been associated with gait velocity (Watson et al., 2010; Morris et al., 2016), therefore suggesting a broad three-way interplay between cognitive function, gait velocity and grey matter volume. It is currently unclear whether gait velocity is directly impacted by cognition, or whether impairments in both gait and cognition are as a result of changes within the brain. Our understanding of the interaction between gait, cognition and the brain, and whether it applies to gait characteristics other than velocity, is limited due to the relative scarcity of studies assessing cognition in addition to gait and neuroimaging parameters.

### 4.6 Current limitations and recommendations for future work

The literature is currently dominated by assessments of gait velocity, most likely due to its ease of measurement. Also, it is frequently assessed alongside other motor parameters in studies assessing “mobility” as opposed to pure gait. Gait velocity is a global measure of gait, which has been assessed through a wide range of techniques; its lack of specificity cannot reflect subtle gait changes that occur during ageing and disease, and its use increases the likelihood of chance findings and different clinical interpretations of results (Graham et al., 2008). There is a relative scarcity of studies assessing gait characteristics other than gait velocity, therefore it cannot be concluded with any certainty whether characteristics within the same gait domain have similar neural underpinnings. Similarities in the grey matter regions associated with gait velocity and step length from the pace domain indicate that these may involve similar mechanisms, although it should be emphasised that this may be due to the high correlation between these characteristics. Until such a time that the neural correlates of several characteristics from each of the gait domains are well understood, to confirm whether characteristics within each domain are controlled in a similar manner, we should not attempt to reduce the number of gait characteristics to be associated with imaging parameters moving forward.

The literature mostly consists of assessments of neural structure rather than function, and the specific regions assessed differed across most studies, suggesting a high degree of heterogeneity. Associations between neuroimaging parameters and gait characteristics were typically conducted through correlational approaches. Correlations were not necessarily reflective of causation and can be affected more strongly by sample size; therefore any identified correlation should be interpreted with some degree of caution. It is justifiable to perform correlational analyses in the first instance, particularly given our limited understanding of the neural control of gait. However, correlations alone do not allow for a strong understanding of the neural control of gait; moving forward it is crucial to develop methodologies which allow for the concurrent measurement of discrete gait characteristics and brain functionality with a good degree of spatial resolution to further our knowledge of gait control.

The mini-mental state examination (MMSE) was most commonly used as an assessment of cognition (where cognitive tests were completed); by using an assessment of global cognition, it is difficult to discern whether the neural substrates associated with different cognitive domains, such as frontal regions with executive function, match those neural areas associated with gait. Confounding factors included in analyses generally differed between studies. Additionally, some papers highlighted results from unadjusted models whereas others only reported results from models including a full set of confounding factors. This may have caused some discrepancy when comparing studies. Gait assessed under dual-task conditions has not been considered, which may have moderated our findings. We feel that a more robust understanding of the influence of cognition on neural gait correlates is required before findings from dual-task protocols can be sufficiently interpreted. A limitation of this review is that we have focussed on the neural correlates of gait in healthy older adults. We could have found a greater number of associations if young or middle-aged adults were included, which may not have been confounded by age. We were confident in restricting our search criteria to older adults, so that we could clearly present the current understanding of gait mechanisms during typical ageing, which should better relate to the mechanisms underpinning gait dysfunction in disease. Nonetheless, it is important to highlight that the mechanisms of gait during earlier life stages may not be fully encompassed by the neural gait correlates presented here.

**Fig. 6:**
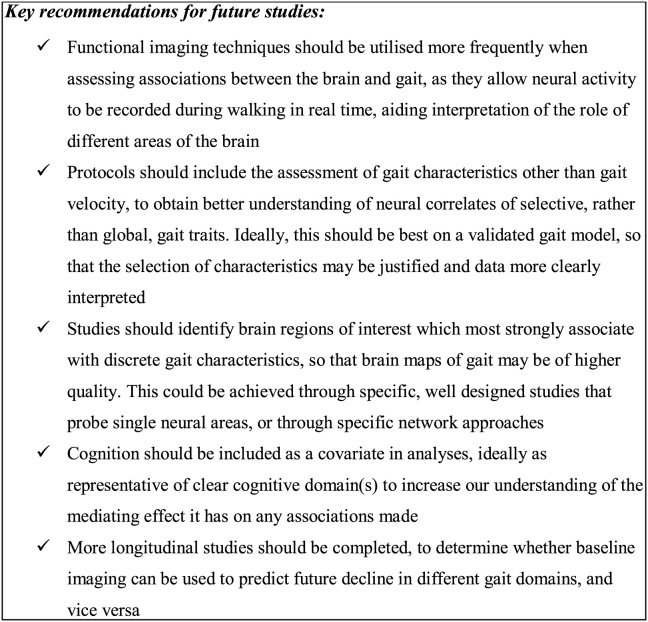
Key recommendations for future studies.

Overall, most of the studies considered within this review were only of average quality (see supplementary Table 1). We have therefore summarised some key recommendations for future studies within this field, see Fig. 6.

## 5 Conclusion

In summary, our structured review has demonstrated that global imaging markers of a ‘deteriorating brain’, namely grey matter atrophy, high volume of white matter hyperintensity lesions, and worsening of diffusion tensor imaging measures of white matter integrity, are correlated with poor gait performance. Additionally, we found that gait velocity decline over time can be predicted by an initial global assessment of imaging markers of white matter. Regionally, we identified a predilection for both the volume of and white matter hyperintensity presence within frontal and basal ganglia regions to influence gait velocity, although many brain regions have been related to gait velocity in some capacity. Beyond this our conclusions are more limited, largely due to the small range of discrete gait characteristics included within each study design, as well as a lack of consistency in the brain regions investigated between studies. We hypothesised that discrete gait characteristics may have more discrete neural correlates; although global associations have been more concretely assessed, there is an emerging specificity of associations between gait and the brain, evidenced by differences in the neural regions that have been associated with different gait characteristics thus far. This review has also demonstrated a relative scarcity of functional imaging correlates with formal gait measures; only one study utilised resting state functional networks in analyses, and only one functional near infra-red spectroscopy study has identified neural gait correlates through single task walking. This is somewhat surprising given the dynamic nature of gait, and given that the association between gait decline and a loss of generalised white matter integrity highlighted in this review points strongly towards an association between gait decline and reduced functional connectivity. Further work should take greater consideration of the covariates included within analyses, and particularly assess the influence of cognition on any associations found, so that a strong model of the three-way interplay between gait, cognition and the brain can be developed.

## Conflicts of interest/disclosure statement

The authors have no conflict of interest to report.
