# Supplementary material for: The neural correlates of discrete gait characteristics in ageing: A structured review
Source: Neurosci Biobehav Rev. 2019 May;100:344–69. doi: 10.1016/j.neubiorev.2018.12.017 (PMC6565843; doi:10.1016/j.neubiorev.2018.12.017)
Supplement: Supplementary file 1 [file mmc1.docx]

**Supplementary table 1.** A quality assessment of all studies included within this review, as completed by reviewers J.W. and R.M.A., in order of largest to smallest study size

| **Study** | **Was the research question or objective in this paper clearly stated?** | **Was the study population clearly specified and defined?** | **Were withdrawals reported and explained?** | **Were inclusion and exclusion criteria for participants defined and determined *prior* to the study onset?** | **Was a sample size justification, power description, or variance and effect estimates provided?** | **Were the gait measures clearly defined, valid, reliable, and implemented consistently across all study participants?** | **Were the image analysis techniques utilized, and the associated imaging parameters, valid, reliable and described in sufficient detail?** | **Were brain regions of interest clearly defined?** | **Were key potential confounding variables measured and their impact on the outcome(s) statistically adjusted for?** | **Quality Assessment: Reviewer 1 (J.W.)** | **Quality Assessment:**  **Reviewer 2 (R.M.A.)** |
| --- | --- | --- | --- | --- | --- | --- | --- | --- | --- | --- | --- |
| **[1] Rosano 2005 (b)** (n=2450) | Yes | Yes | J.W. No  R.M.A. Yes | Yes | No | No | No | No | Yes | Average (4/9) | Average (5/9) |
| **[2] Verlinden 2016** (n=2330) | Yes | No | Yes | No | No | Yes | Yes | Yes | Yes | Average (6/9) | Average (6/9) |
| **[3] Soumaré 2009** (n=1702) | Yes | Yes | No | Yes | No | Yes | Yes | Yes | Yes | Good (7/9) | Good (7/9) |
| **[4] Dumurgier 2012** (n=1623) | Yes | Yes | Yes | Yes | No | Yes | Yes | Yes | Yes | Good (8/9) | Good (8/9) |
| **[5] Willey 2013** (n=701) | Yes | Yes | No | No | No | No | Yes | J.W. Yes  R.M.A. No | Yes | Average (5/9) | Average (4/9) |
| **[6] Baezner 2008** (n=639) | Yes | J.W. Yes  R.M.A. No | No | Yes | No | J.W. No  R.M.A. Yes | No | No | Yes | Average (4/9) | Average (4/9) |
| **[7] Wennberg 2017** (n=611) | Yes | Yes | No | Yes | No | Yes | Yes | Yes | Yes | Good (7/9) | Good (7/9) |
| **[8] Ryberg 2011** (n=563) | Yes | Yes | No | Yes | No | No | Yes | Yes | Yes | Average (6/9) | Average (6/9) |
| **[9] De Laat 2011 (c)** (n=485) | Yes | Yes | No | No | No | Yes | Yes | Yes | Yes | Average (6/9) | Average (6/9) |
| **[10] De Laat 2011 (a)** (n=484) | Yes | Yes | Yes | Yes | No | Yes | Yes | Yes | Yes | Good (8/9) | Good (8/9) |
| **[11] De Laat 2011 (b)** (n=429) | Yes | Yes | Yes | Yes | No | Yes | Yes | Yes | Yes | Good (8/9) | Good (8/9) |
| **[12] De Laat 2012** (n=415) | Yes | Yes | Yes | Yes | No | Yes | Yes | Yes | Yes | Good (8/9) | Good (8/9) |
| **[13] Choi 2012** (n=377) | Yes | Yes | J.W. No  R.M.A. Yes | Yes | No | Yes | Yes | No | Yes | Average (6/9) | Good (7/9) |
| **[14] Holtzer 2015** (n=348) | Yes | Yes | No | Yes | No | Yes | Yes | Yes | Yes | Good (7/9) | Good (7/9) |
| **[15] Rosano 2007 (b)** (n=331) | Yes | Yes | No | Yes | No | Yes | No | Yes | Yes | Average (6/9) | Average (6/9) |
| **[16] Fredriksen 2011** (n=328) | Yes | Yes | J.W. No  R.M.A. Yes | Yes | No | No | Yes | Yes | No | Average (5/9) | Average (6/9) |
| **[17] Rosano 2007 (a)** (n=327) | Yes | Yes | No | No | No | No | Yes | Yes | Yes | Average (5/9) | Average (5/9) |
| **[18] Rosano 2005 (a)** (n=321) | Yes | Yes | No | Yes | No | Yes | No | Yes | Yes | Average (6/9) | Average (6/9) |
| **[19] Callisaya 2014** (n=305) | Yes | Yes | No | Yes | No | Yes | Yes | Yes | Yes | Good (7/9) | Good (7/9) |
| **[20] van der Holst 2018 (n=275)** | Yes | Yes | Yes | Yes | No | Yes | Yes | Yes | Yes | Good (8/9) | Good (8/9) |
| **[21] Rosso 2014** (n=265) | Yes | Yes | No | Yes | No | Yes | Yes | Yes | Yes | Good (7/9) | Good (7/9) |
| **[22] Rosario 2016** (n=265) | Yes | Yes | No | Yes | No | Yes | Yes | Yes | Yes | Good (7/9) | Good (7/9) |
| **[23] Bolandzadeh 2014** (n=253) | Yes | Yes | Yes | Yes | No | No | Yes | No | Yes | Average (6/9) | Average (6/9) |
| **[24] Stijntjes 2016** (n=237) | No | Yes | No | No | No | No | Yes | Yes | Yes | Average (4/9) | Average (4/9) |
| **[25] Nadkarni 2014** (n=231) | Yes | Yes | J.W. No  R.M.A. Yes | Yes | No | Yes | Yes | Yes | Yes | Good (7/9) | Good (8/9) |
| **[26] Callisaya 2013** (n=225) | Yes | Yes | Yes | Yes | No | Yes | Yes | Yes | Yes | Good (8/9) | Good (8/9) |
| **[27] Rosano 2008** (n=220) | Yes | Yes | No | No | No | Yes | Yes | Yes | Yes | Average (6/9) | Average (6/9) |
| **[28] Nadkarni 2017** (n=183) | Yes | J.W. No  R.M.A. Yes | Yes | No | No | Yes | Yes | Yes | Yes | Average (6/9) | Good (7/9) |
| **[29] Sakurai 2014** (n=182) | Yes | Yes | No | Yes | No | No | Yes | Yes | Yes | Average (5/9) | Average (6/9) |
| **[30] Sakurai 2017** (n=149) | Yes | Yes | No | Yes | No | No | Yes | Yes | Yes | Average (6/9) | Average (6/9) |
| **[31] Murray 2010** (n=148) | Yes | Yes | No | Yes | No | Yes | Yes | Yes | No | Average (6/9) | Average (6/9) |
| **[32] del Campo 2016** (n=128) | Yes | J.W. No  R.M.A. Yes | Yes | Yes | No | No | Yes | Yes | Yes | Average (6/9) | Good (7/9) |
| **[33] Annweiler 2014** (n=115) | Yes | No | Yes | Yes | No | Yes | Yes | Yes | Yes | Good (7/9) | Good (7/9) |
| **[34] Ezzati 2015** (n=112) | Yes | Yes | Yes | Yes | No | Yes | Yes | Yes | Yes | Good (8/9) | Good (8/9) |
| **[35] Manor 2012** (n=89) | Yes | Yes | No | Yes | No | Yes | Yes | Yes | Yes | Good (7/9) | Good (7/9) |
| **[36] Beauchet 2017** (n=77) | Yes | Yes | No | Yes | No | Yes | Yes | Yes | Yes | Good (7/9) | Good (7/9) |
| **[37] Moscufo 2012** (n=77) | Yes | Yes | Yes | Yes | No | No | Yes | Yes | Yes | Good (7/9) | Good (7/9) |
| **[38] Novak 2009** (n=76) | Yes | Yes | No | Yes | No | J.W. No  R.M.A. Yes | Yes | Yes | Yes | Average (6/9) | Good (7/9) |
| **[39] Beauchet 2014** (n=71) | Yes | No | No | Yes | No | Yes | Yes | Yes | Yes | Average (6/9) | Average (6/9) |
| **[40] Wolfson 2013** (n=67) | Yes | Yes | Yes | Yes | No | No | Yes | No | Yes | Average (6/9) | Average (6/9) |
| **[41] Tian 2017** (n=59) | Yes | Yes | No | Yes | No | No | Yes | Yes | Yes | Average (6/9) | Average (6/9) |
| **[42] Zimmerman 2009** (n=48) | Yes | Yes | No | No | No | Yes | Yes | Yes | Yes | Average (6/9) | Average (6/9) |
| **[43] Beauchet 2015** (n=47) | Yes | Yes | No | Yes | No | Yes | Yes | Yes | Yes | Good (7/9) | Good (7/9) |
| **[44] Sorond 2011** (n=42) | Yes | Yes | No | Yes | No | No | No | No | No | Poor (3/9) | Poor (3/9) |
| **[45] Della Nave 2007** (n=36) | J.W. No  R.M.A. Yes | Yes | No | No | No | No | Yes | Yes | No | Poor (3/9) | Average (4/9) |
| **[46] Nadkarni 2009** (n=33) | Yes | No | No | Yes | No | Yes | No | Yes | No | Average (4/9) | Average (4/9) |
| **[47] Yuan 2015** (n=30) | Yes | J.W. Yes  R.M.A. No | No | Yes | No | Yes | Yes | Yes | No | Average (6/9) | Average (5/9) |
| **[48] Bruijn 2014** (n=25) | Yes | No | No | No | No | Yes | Yes | Yes | No | Average (4/9) | Average (4/9) |
| **[49] Shimada 2013** (n=24) | Yes | No | No | Yes | No | Yes | Yes | Yes | No | Average (5/9) | Average (5/9) |
| **[50] Fling 2018** (n=20) | Yes | Yes | No | No | No | Yes | Yes | Yes | No | Average (5/9) | Average (5/9) |
| **[51] Wolfson 2005** (n=14) | Yes | No | No | Yes | No | No | Yes | No | No | Poor (3/9) | Poor (3/9) |
| **[52] Fling 2016** (n=10) | Yes | Yes | No | Yes | No | Yes | Yes | Yes | Yes | Good (7/9) | Good (7/9) |
| **TOTAL** |  | | | | | | | | | 20 Good | 24 Good |
|  |  |  |  |  |  |  |  |  |  | 29 Average | 26 Average |
|  |  |  |  |  |  |  |  |  |  | 3 Poor | 2 Poor |

**Supplementary table 2**. Definitions of common gait terms

| **Gait term** | **Definition** |
| --- | --- |
| **Step** | Where one leg moves forward during walking |
| **Stride** | Where both a left and right footstep have been taken |
| **Velocity** | The speed of walking, typically in units of metres per second (ms^-1^) |
| **Step length** | The distance between the heels of both feet once a step has been taken, typically measured in metres (m) |
| **Step time** | The time taken to make a step, typically measured in seconds (s) |
| **Stride time** | The time taken to make a stride (s), which can also be referred to as *gait cycle duration* |
| **Swing time** | The time during which a foot is off the ground (s) |
| **Stance time** | The time during which a foot is on the ground (s), which can also be referred to as single support time or phase |
| **Double support** | Where both feet are on the ground during walking |
| **Cadence** | The number of steps taken during a defined time measure, for example, steps per minute. This is the inverse of step time |
| **Step width** | The mediolateral distance between heels during double support (m) |
| **Variability** | Refers to step-to-step variations in spatio-temporal gait measures |
| **Asymmetry** | Refers to the ratio between left and right steps |

**Supplementary table 3.** Summary of cross-sectional associations between parameters from MRI volumetric analysis and gait characteristics. Green indicates a positive association was found, red indicates a negative association found and yellow indicates that no association was found. Numbers refer to supplementary references

|  | | **MRI – volumetric analysis** | | | |
| --- | --- | --- | --- | --- | --- |
| **Gait domain** | **Gait characteristic** | **GM volume** | **WM volume** | **Total brain volume** | **Ventricular volume** |
| **Pace** | **Gait velocity** | **[4] [12] [17] [19] [24] [25] [34] [38]** | **[38]** | **[10]** |  |
|  |  | **[24] [35] [36] [45]** | **[19] [24] [34] [45]** | **[44] [45]** | **[18] [34]** |
|  |  |  |  |  | **[1]** |
|  | **Step length** | **[12] [19] [27] [42]** |  | **[10]** |  |
|  |  |  | **[19]** |  | **[18]** |
|  |  |  |  |  |  |
|  | **Step time variability** | **[43]** |  |  | **[33]** |
|  |  | **[35] [36]** | **[39]** |  |  |
|  |  | **[39]** |  | **[10]** |  |
|  | **Swing time variability** |  |  |  |  |
|  |  | **[43]** |  |  |  |
|  |  |  |  |  |  |
| **Rhythm** | **Double support time** |  |  |  |  |
|  |  | **[35]** |  |  | **[18]** |
|  |  | **[27]** |  | **[10]** |  |
|  | **Cadence** | **[12]** |  | **[10]** |  |
|  |  | **[19]** | **[19]** |  |  |
|  |  |  |  |  |  |
|  | **Swing time** |  |  |  |  |
|  |  | **[43]** |  |  |  |
|  |  |  |  |  |  |
|  | **Step time** |  |  |  |  |
|  |  | **[43]** |  |  |  |
|  |  |  |  |  |  |
| **Variability** | **Step length variability** |  |  |  |  |
|  |  | **[21]** |  |  |  |
|  |  |  |  | **[10]** |  |
|  | **Step width variability** |  |  |  |  |
|  |  | **[43]** |  | **[10]** |  |
|  |  |  |  |  |  |
| **Postural Control** | **Step width** |  |  |  |  |
|  |  | **[43]** |  |  |  |
|  |  | **[12] [27]** |  | **[10]** |  |

**Supplementary table 4.** Summary of cross-sectional associations between white matter changes associated with age, from MRI, and gait characteristics. Green indicates a positive association was found, red indicates a negative association found and yellow indicates that no association was found. Numbers refer to supplementary references.

|  | | **MRI – white matter changes associated with age** | | |
| --- | --- | --- | --- | --- |
| **Gait domain** | **Gait characteristic** | **WMH presence** | **Infarct presence** | **Microbleed presence** |
| **Pace** | **Gait velocity** |  |  |  |
|  |  | **[24] [38] [45]** |  | **[13]** |
|  |  | **[1] [3] [5] [6] [11] [18] [22] [23] [31] [37] [40] [44] [46]** | **[1] [13] [18] [24]** | **[9] [24]** |
|  | **Step length** |  |  |  |
|  |  | **[18] [46]** |  | **[13]** |
|  |  | **[11]** | **[13] [18]** | **[9]** |
|  | **Stance time variability** | **[15]** |  |  |
|  |  |  | **[15]** |  |
|  |  |  |  |  |
| **Rhythm** | **Double support time** | **[18]** | **[13]** | **[9] [13]** |
|  |  |  | **[18]** |  |
|  |  |  |  |  |
|  | **Cadence** |  |  |  |
|  |  | **[11] [46]** | **[13]** | **[9]** |
|  |  |  |  | **[13]** |
| **Variability** | **Step length variability** | **[15]** | **[15]** |  |
|  |  | **[21]** |  |  |
|  |  |  |  |  |
|  | **Step width variability** |  |  |  |
|  |  | **[15]** | **[15]** |  |
|  |  |  |  |  |
| **Postural Control** | **Step width** | **[11]** | **[13]** | **[9]** |
|  |  |  |  | **[13]** |
|  |  | **[46]** |  |  |

**Supplementary table 5**. Summary of cross-sectional associations between parameters from DTI and gait characteristics. Green indicates a positive association was found, red indicates a negative association found and yellow indicates that no association was found. Numbers refer to supplementary references

|  | | **DTI** | | | |
| --- | --- | --- | --- | --- | --- |
| **Gait domain** | **Gait characteristic** | **FA** | **MD** | **RD** | **AD** |
| **Pace** | **Gait velocity** | **[2] [10] [11]** |  |  |  |
|  |  | **[45] [52]** | **[45]** |  |  |
|  |  |  | **[2] [10] [11]** | **[2] [11]** | **[2] [11]** |
|  | **Step length** | **[10] [11]** |  |  |  |
|  |  |  |  |  |  |
|  |  |  | **[2] [10] [11]** | **[11]** | **[11]** |
|  | **Step time variability** |  | **[10]** |  |  |
|  |  |  |  |  |  |
|  |  | **[10]** |  |  |  |
| **Rhythm** | **Double support time** |  | **[10]** |  |  |
|  |  | **[10]** |  |  |  |
|  |  |  |  |  |  |
|  | **Cadence** | **[10] [11]** |  |  |  |
|  |  |  |  |  |  |
|  |  |  | **[10] [11]** | **[11]** | **[11]** |
|  | **Stance time** |  |  |  |  |
|  |  |  | **[2]** |  |  |
| **Variability** | **Step length variability** |  | **[2] [10] [21]** |  |  |
|  |  | **[10]** |  |  |  |
|  |  | **[21]** |  |  |  |
|  | **Step width variability** |  |  |  |  |
|  |  | **[10]** | **[10]** |  |  |
|  |  |  |  |  |  |
| **Asymmetry** | **Step time asymmetry** |  |  |  |  |
|  |  | **[50]** |  |  |  |
|  |  |  |  |  |  |
| **Postural Control** | **Step width** | **[48]** | **[10] [11]** | **[11]** | **[11]** |
|  |  | **[52]** |  |  |  |
|  |  | **[10] [11]** |  |  |  |
|  | **Step length asymmetry** |  |  |  |  |
|  |  | **[50]** |  |  |  |
|  |  |  |  |  |  |

**Supplementary table 6.** Summary of cross-sectional associations between parameters from PET and gait characteristics. Green indicates a positive association was found, red indicates a negative association found and yellow indicates that no association was found. Numbers refer to supplementary references

|  | | **PET** | | | |
| --- | --- | --- | --- | --- | --- |
| **Gait domain** | **Gait characteristic** | **Aβ burden** | **Glucose uptake** | | |
| **Pace** | **Gait velocity** |  | **[29] [30]** | | |
|  |  | **[41]** |  | | |
|  |  | **[7] [28] [32]** |  |  |  |
|  | **Step length** |  |  | | |
|  |  | **[7]** | **[29] [30]** | | |
|  |  |  |  | | |
|  | **Stance time variability** | **[7]** |  | | |
|  |  |  |  |  |  |
| **Rhythm** | **Double support time** | **[7]** |  | | |
|  |  |  |  |  |  |
|  | **Cadence** |  | **[29] [30]** | | |
|  |  |  |  | | |
|  |  | **[7]** |  |  |  |
| **Variability** | **Step length variability** |  |  |  |  |
|  |  |  |  | | |
|  |  |  | **[49]** | | |

**Supplementary table 7**. Summary of longitudinal associations between imaging parameters and gait characteristics. Green indicates a positive association was found, red indicates a negative association found and yellow indicates that no association was found. Numbers refer to supplementary references. * indicates that baseline image parameters predicted changes in gait; where there is no symbol, changes in imaging parameters related to changes in gait.

|  | | **Longitudinal Imaging** | | | | | | | | | | |
| --- | --- | --- | --- | --- | --- | --- | --- | --- | --- | --- | --- | --- |
|  | | **MRI – volumetric analysis** | | | | **MRI – white matte changes associated with age** | | | **DTI** | | | **PET** |
| **Gait domain** | **Gait characteristic** | **Grey matter volume** | **White matter volume** | **CSF volume** | **Ventricle volume** | **WMH presence** | **Infarct presence** | **Microbleed presence** | **FA** | **MD & RD** | **AD** | **Aβ burden** |
| **Pace** | **Gait velocity** | **[26]** | **[8*] [26] [51*]** |  |  |  |  |  |  |  |  |  |
|  |  | **[20]** | **[16] [20]** |  |  | **[20] [37] [40*]** | **[20]** | **[20]** | **[20]** | **[20]** | **[20]** |  |
|  |  |  |  | **[51*]** | **[1*]** | **[1*] [3*] [5*] [26]** | **[1*]** |  |  |  |  | **[41*]** |
|  | **Step length** | **[26]** | **[20] [26]** |  |  |  |  |  | **[20]** |  |  |  |
|  |  | **[20] [26]** |  |  |  | **[20]** | **[20]** | **[20]** |  |  | **[20]** |  |
|  |  |  |  |  |  | **[26]** |  |  |  | **[20]** |  |  |
| **Rhythm** | **Cadence** | **[26]** | **[26]** |  |  |  |  |  |  |  |  |  |
|  |  | **[20] [26]** | **[20]** |  |  | **[20] [26]** | **[20]** | **[20]** | **[20]** | **[20]** | **[20]** |  |
|  |  |  |  |  |  |  |  |  |  |  |  |  |
| **Postural Control** | **Step width** |  |  |  |  |  |  |  |  |  |  |  |
|  |  | **[26]** | **[26]** |  |  | **[26]** |  |  |  |  |  |  |
|  |  |  |  |  |  |  |  |  |  |  |  |  |

**References for supplementary information**

[1] Rosano, C., Kuller, L.H., Chung, H., Arnold, A.M., Longstreth Jr, W.T. and Newman, A.B. (2005b) 'Subclinical brain magnetic resonance imaging abnormalities predict physical functional decline in high-functioning older adults', Journal of the American Geriatrics Society, 53(4), pp. 649-654.

[2] Verlinden, V.J., de Groot, M., Cremers, L.G., van der Geest, J.N., Hofman, A., Niessen, W.J., van der Lugt, A., Vernooij, M.W. and Ikram, M. (2016) 'Tract-specific white matter microstructure and gait in humans', Neurobiology of Aging, 43, pp. 164-173.

[3] Soumaré, A., Elbaz, A., Zhu, Y., Maillard, P., Crivello, F., Tavernier, B., Dufouil, C., Mazoyer, B. and Tzourio, C. (2009) 'White matter lesions volume and motor performances in the elderly', Annals of Neurology, 65(6), pp. 706-715.

[4] Dumurgier, J., Crivello, F., Mazoyer, B., Ahmed, I., Tavernier, B., Grabli, D., François, C., Tzourio-Mazoyer, N., Tzourio, C. and Elbaz, A. (2012) 'MRI atrophy of the caudate nucleus and slower walking speed in the elderly', NeuroImage, 60(2), pp. 871-878.

[5] Willey, J.Z., Scarmeas, N., Provenzano, F.A., Luchsinger, J.A., Mayeux, R. and Brickman, A.M. (2013) 'White matter hyperintensity volume and impaired mobility among older adults', Journal of Neurology, 260(3), pp. 884-890.

[6] Baezner, H., Blahak, C., Poggesi, A., Pantoni, L., Inzitari, D., Chabriat, H., Erkinjuntti, T., Fazekas, F., Ferro, J.M., Langhorne, P., O'Brien, J., Scheltens, P., Visser, M.C., Wahlund, L.O., Waldemar, G., Wallin, A. and Hennerici, M.G. (2008) 'Association of gait and balance disorders with age-related white matter changes: The LADIS Study', Neurology, 70(12), pp. 935-942.

[7] Wennberg, A.M.V., Savica, R., Hagen, C.E., Roberts, R.O., Knopman, D.S., Hollman, J.H., Vemuri, P., Jack, C.R., Petersen, R.C. and Mielke, M.M. (2017) 'Cerebral Amyloid Deposition Is Associated with Gait Parameters in the Mayo Clinic Study of Aging', Journal of the American Geriatrics Society, 65(4), pp. 792-799.

[8] Ryberg, C., Rostrup, E., Paulson, O.B., Barkhof, F., Scheltens, P., Van Straaten, E.C.W., Van Der Flier, W.M., Fazekas, F., Schmidt, R., Ferro, J.M., Baezner, H., Erkinjuntti, T., Jokinen, H., Wahlund, L.O., Poggesi, A., Pantoni, L., Inzitari, D. and Waldemar, G. (2011) 'Corpus callosum atrophy as a predictor of age-related cognitive and motor impairment: A 3-year follow-up of the LADIS study cohort', Journal of the Neurological Sciences, 307(1-2), pp. 100-105.

[9] de Laat, K.F., Van Norden, A.G.W., Gons, R.A.R., Van Oudheusden, L.J.B., Van Uden, I.W.M., Norris, D.G., Zwiers, M.P. and De Leeuw, F.E. (2011c) 'Diffusion tensor imaging and gait in elderly persons with cerebral small vessel disease', Stroke, 42(2), pp. 373-379.

[10] de Laat, K.F., Tuladhar, A.M., van Norden, A.G., Norris, D.G., Zwiers, M.P. and de Leeuw, F.-E. (2011a) 'Loss of White matter integrity is associated with gait disorders in cerebral small vessel disease', Brain: A Journal of Neurology, 134(1), pp. 73-83.

[11] de Laat, K.F., Van Den Berg, H.A.C., Van Norden, A.G.W., Gons, R.A.R., Olde Rikkert, M.G.M. and De Leeuw, F.E. (2011b) 'Microbleeds are independently related to gait disturbances in elderly individuals with cerebral small vessel disease', Stroke, 42(2), pp. 494-497.

[12] de Laat, K.F., Reid, A.T., Grim, D.C., Evans, A.C., Kotter, R., van Norden, A.G. and de Leeuw, F.-E. (2012) 'Cortical thickness is associated with gait disturbances in cerebral small vessel disease', NeuroImage, 59(2), pp. 1478-1484.

[13] Choi, P., Ren, M., Phan, T.G., Callisaya, M., Ly, J.V., Beare, R., Chong, W. and Srikanth, V. (2012) 'Silent infarcts and cerebral microbleeds modify the associations of white matter lesions with gait and postural stability: Population-based study', Stroke, 43(6), pp. 1505-1510.

[14] Holtzer, R., Mahoney, J.R., Izzetoglu, M., Wang, C., England, S. and Verghese, J. (2015) 'Online fronto-cortical control of simple and attention-demanding locomotion in humans', NeuroImage, 112, pp. 152-159.

[15] Rosano, C., Brach, J., Studenski, S., Longstreth, W. and Newman, A.B. (2007b) 'Gait variability is associated with subclinical brain vascular abnormalities in high-functioning older adults', Neuroepidemiology, 29(3-4), pp. 193-200.

[16] Frederiksen, K.S., Garde, E., Skimminge, A., Barkhof, F., Scheltens, P., van Straaten, E.C., Fazekas, F., Baezner, H., Verdelho, A., Ferro, J.M., Erkinjuntti, T., Jokinen, H., Wahlund, L.O., O'Brien, J.T., Basile, A.M., Pantoni, L., Inzitari, D. and Waldemar, G. (2011) 'Corpus callosum tissue loss and development of motor and global cognitive impairment: the LADIS study', Dementia & Geriatric Cognitive Disorders, 32(4), pp. 279-86.

[17] Rosano, C., Aizenstein, H.J., Studenski, S. and Newman, A.B. (2007a) 'A regions-of-interest volumetric analysis of mobility limitations in community-dwelling older adults', Journals of Gerontology - Series A Biological Sciences and Medical Sciences, 62(9), pp. 1048-1055.

[18] Rosano, C., Brach, J., Longstreth Jr, W.T. and Newman, A.B. (2005a) 'Quantitative measures of gait characteristics indicate prevalence of underlying subclinical structural brain abnormalities in high-functioning older adults', Neuroepidemiology, 26(1), pp. 52-60.

[19] Callisaya, M.L., Beare, R., Phan, T.G., Chen, J. and Srikanth, V.K. (2014) 'Global and regional associations of smaller cerebral gray and white matter volumes with gait in older people', PLoS ONE, 9(1).

[20] van der Holst, H.M., Tuladhar, A.M., Zerbi, V., van Uden, I.W.M., de Laat, K.F., van Leijsen, E.M.C., Ghafoorian, M., Platel, B., Bergkamp, M.I., van Norden, A.G.W., Norris, D.G., van Dijk, E.J., Kiliaan, A.J. and de Leeuw, F.E. (2018) 'White matter changes and gait decline in cerebral small vessel disease', NeuroImage : Clinical, 17, pp. 731-738.

[21] Rosso, A.L., Olson Hunt, M.J., Yang, M., Brach, J.S., Harris, T.B., Newman, A.B., Satterfield, S., Studenski, S.A., Yaffe, K., Aizenstein, H.J. and Rosano, C. (2014) 'Higher step length variability indicates lower gray matter integrity of selected regions in older adults', Gait and Posture, 40(1), pp. 225-230.

[22] Rosario, B.L., Rosso, A.L., Aizenstein, H.J., Harris, T., Newman, A.B., Satterfield, S., Studenski, S.A., Yaffe, K. and Rosano, C. (2016) 'Cerebral white matter and slow gait: Contribution of hyperintensities and normal-appearing parenchyma', Journals of Gerontology - Series A Biological Sciences and Medical Sciences, 71(7), pp. 968-973.

[23] Bolandzadeh, N., Liu-Ambrose, T., Aizenstein, H., Harris, T., Launer, L., Yaffe, K., Kritchevsky, S.B., Newman, A. and Rosano, C. (2014) 'Pathways linking regional hyperintensities in the brain and slower gait', NeuroImage, 99, pp. 7-13.

[24] Stijntjes, M., De Craen, A.J.M., Van Der Grond, J., Meskers, C.G.M., Slagboom, P.E. and Maier, A.B. (2016) 'Cerebral microbleeds and lacunar infarcts are associated with walking speed independent of cognitive performance in middle-aged to older adults', Gerontology, 62(5), pp. 500-507.

[25] Nadkarni, N.K., Nunley, K.A., Aizenstein, H., Harris, T.B., Yaffe, K., Satterfield, S., Newman, A.B. and Rosano, C. (2014) 'Association between cerebellar gray matter volumes, gait speed, and information-processing ability in older adults enrolled in the Health ABC study', The Journals of Gerontology: Series A: Biological Sciences and Medical Sciences, 69(8), pp. 996-1003.

[26] Callisaya, M.L., Beare, R., Phan, T.G., Blizzard, L., Thrift, A.G., Chen, J. and Srikanth, V.K. (2013) 'Brain structural change and gait decline: A longitudinal population-based study', Journal of the American Geriatrics Society, 61(7), pp. 1074-1079.

[27] Rosano, C., Aizenstein, H., Brach, J., Longenberger, A., Studenski, S. and Newman, A.B. (2008) 'Gait measures indicate underlying focal gray matter atrophy in the brain of older adults', Journals of Gerontology - Series A Biological Sciences and Medical Sciences, 63(12), pp. 1380-1388.

[28] Nadkarni, N.K., Perera, S., Snitz, B.E. and et al. (2017) 'Association of brain amyloid-β with slow gait in elderly individuals without dementia: Influence of cognition and apolipoprotein e ε4 genotype', JAMA Neurology, 74(1), pp. 82-90.

[29] Sakurai, R., Fujiwara, Y., Yasunaga, M., Takeuchi, R., Murayama, Y., Ohba, H., Sakuma, N., Suzuki, H., Oda, K., Sakata, M., Toyohara, J., Ishiwata, K., Shinkai, S. and Ishii, K. (2014) 'Regional cerebral glucose metabolism and gait speed in healthy community-dwelling older women', Journals of Gerontology - Series A Biological Sciences and Medical Sciences, 69(12), pp. 1519-1527.

[30] Sakurai, R., Ishii, K., Yasunaga, M., Takeuchi, R., Murayama, Y., Sakuma, N., Sakata, M., Oda, K., Ishibashi, K., Ishiwata, K., Fujiwara, Y. and Montero-Odasso, M. (2017) 'The neural substrate of gait and executive function relationship in elderly women: A PET study', Geriatrics and Gerontology International.

[31] Murray, M.E., Senjem, M.L., Petersen, R.C., Hollman, J.H., Preboske, G.M., Weigand, S.D., Knopman, D.S., Ferman, T.J., Dickson, D.W. and Jack, C.R., Jr. (2010) 'Functional impact of white matter hyperintensities in cognitively normal elderly subjects', Archives of Neurology, 67(11), pp. 1379-1385.

[32] del Campo, N., Payoux, P., Djilali, A., Delrieu, J., Hoogendijk, E.O., Rolland, Y., Cesari, M., Weiner, M.W., Andrieu, S. and Vellas, B. (2016) 'Relationship of regional brain beta-amyloid to gait speed', Neurology, 86(1), pp. 36-43.

[33] Annweiler, C., Montero-Odasso, M., Bartha, R., Drozd, J., Hachinski, V. and Beauchet, O. (2014) 'Association between gait variability and brain ventricle attributes: A brain mapping study', Experimental Gerontology, 57, pp. 256-263.

[34] Ezzati, A., Katz, M.J., Lipton, M.L., Lipton, R.B. and Verghese, J. (2015) 'The association of brain structure with gait velocity in older adults: a quantitative volumetric analysis of brain MRI', Neuroradiology, 57(8), pp. 851-861.

[35] Manor, B., Newton, E., Abduljalil, A. and Novak, V. (2012) 'The Relationship Between Brain Volume and Walking Outcomes in Older Adults With and Without Diabetic Peripheral Neuropathy', Diabetes Care, 35(9), pp. 1907-1912.

[36] Beauchet, O., Launay, C.P., Barden, J., Liu-Ambrose, T., Chester, V.L., Szturm, T., Grenier, S., Leonard, G., Bherer, L., Annweiler, C., Helbostad, J.L., Verghese, J. and Allali, G. (2017) 'Association between falls and brain subvolumes: Results from a cross-sectional analysis in healthy older adults', Brain Topography, 30(2), pp. 272-280.

[37] Moscufo, N., Wolfson, L., Meier, D., Liguori, M., Hildenbrand, P.G., Wakefield, D., Schmidt, J.A., Pearlson, G.D. and Guttmann, C.R.G. (2012) 'Mobility decline in the elderly relates to lesion accrual in the splenium of the corpus callosum', Age, 34(2), pp. 405-414.

[38] Novak, V., Haertle, M., Zhao, P., Hu, K., Munshi, M., Novak, P., Abduljalil, A. and Alsop, D. (2009) 'White matter hyperintensities and dynamics of postural control', Magnetic Resonance Imaging, 27(6), pp. 752-759.

[39] Beauchet, O., Annweiler, C., Celle, S., Bartha, R., Barthelemy, J.-C. and Roche, F. (2014) 'Higher gait variability is associated with decreased parietal gray matter volume among healthy older adults', Brain Topography, 27(2), pp. 293-295.

[40] Wolfson, L., Wakefield, D.B., Moscufo, N., Kaplan, R.F., Hall, C.B., Schmidt, J.A., Guttmann, C.R.G. and White, W.B. (2013) 'Rapid buildup of brain white matter hyperintensities over 4 years linked to ambulatory blood pressure, mobility, cognition, and depression in old persons', Journals of Gerontology - Series A Biological Sciences and Medical Sciences, 68(11), pp. 1387-1394.

[41] Tian, Q., Resnick, S.M., Bilgel, M., Wong, D.F., Ferrucci, L. and Studenski, S.A. (2017) 'beta-Amyloid Burden Predicts Lower Extremity Performance Decline in Cognitively Unimpaired Older Adults', Journals of Gerontology Series A-Biological Sciences & Medical Sciences, 72(5), pp. 716-723.

[42] Zimmerman, M.E., Lipton, R.B., Pan, J.W., Hetherington, H.P. and Verghese, J. (2009) 'MRI- and MRS-derived hippocampal correlates of quantitative locomotor function in older adults', Brain Research, 1291, pp. 73-81.

[43] Beauchet, O., Launay, C.P., Annweiler, C. and Allali, G. (2015) 'Hippocampal volume, early cognitive decline and gait variability: Which association?', Experimental Gerontology, 61, pp. 98-104.

[44] Sorond, F.A., Kiely, D.K., Galica, A., Moscufo, N., Serrador, J.M., Iloputaife, I., Egorova, S., Dell'Oglio, E., Meier, D.S., Newton, E., Milberg, W.P., Guttmann, C.R. and Lipsitz, L.A. (2011) 'Neurovascular coupling is impaired in slow walkers: The MOBILIZE Boston study', Annals of Neurology, 70(2), pp. 213-220.

[45] Della Nave, R., Foresti, S., Pratesi, A., Ginestroni, A., Inzitari, M., Salvadori, E., Giannelli, M., Diciotti, S., Inzitari, D. and Mascalchi, M. (2007) 'Whole-brain histogram and voxel-based analyses of diffusion tensor imaging in patients with leukoaraiosis: Correlation with motor and cognitive impairment', American Journal of Neuroradiology, 28(7), pp. 1313-1319.

[46] Nadkarni, N.K., McIlroy, W.E., Mawji, E. and Black, S.E. (2009) 'Gait and subcortical hyperintensities in mild alzheimer's disease and aging', Dementia and Geriatric Cognitive Disorders, 28(4), pp. 295-301.

[47] Yuan, J., Blumen, H.M., Verghese, J. and Holtzer, R. (2015) 'Functional connectivity associated with gait velocity during walking and walking-while-talking in aging: A resting-state fMRI study', Human Brain Mapping, 36(4), pp. 1484-1493.

[48] Bruijn, S.M., Van Impe, A., Duysens, J. and Swinnen, S.P. (2014) 'White matter microstructural organization and gait stability in older adults', Frontiers in Aging Neuroscience, 6(JUN).

[49] Shimada, H., Ishii, K., Ishiwata, K., Oda, K., Suzukawa, M., Makizako, H., Doi, T. and Suzuki, T. (2013) 'Gait adaptability and brain activity during unaccustomed treadmill walking in healthy elderly females', Gait and Posture, 38(2), pp. 203-208.

[50] Fling, B.W., Curtze, C. and Horak, F.B. (2018) 'Gait Asymmetry in People With Parkinson’s Disease Is Linked to Reduced Integrity of Callosal Sensorimotor Regions', Frontiers in Neurology, 9(215).

[51] Wolfson, L., Wei, X., Hall, C.B., Panzer, V., Wakefield, D., Benson, R.R., Schmidt, J.A., Warfield, S.K. and Guttmann, C.R.G. (2005) 'Accrual of MRI white matter abnormalities in elderly with normal and impaired mobility', Journal of the Neurological Sciences, 232(1-2), pp. 23-27.

[52] Fling, B.W., Dale, M.L., Curtze, C., Smulders, K., Nutt, J.G. and Horak, F.B. (2016) 'Associations between mobility, cognition and callosal integrity in people with parkinsonism', NeuroImage: Clinical, 11, pp. 415-422.
